# A Whole-Brain Cell-Type-Specific Sparse Neuron Labeling Method and Its Application in a *Shank3* Autistic Mouse Model

**DOI:** 10.3389/fncel.2020.00145

**Published:** 2020-06-05

**Authors:** Di Chen, Keke Ren, Haiying Liu, Honghui Mao, Zongyan Li, Huiming Mo, Shengjun Xie, Yiwu Shi, Qian Chen, Wenting Wang

**Affiliations:** ^1^Institute of Neuroscience, Department of Neurology, The Second Affiliated Hospital of Guangzhou Medical University, Guangzhou, China; ^2^Department of Neurobiology, School of Basic Medicine, Fourth Military Medical University, Xi’an, China; ^3^Department of Physiology, Medical College of Yan’an University, Yan’an, China

**Keywords:** sparse neuron labeling, neuronal morphology reconstruction, dendritic spine, autism, CaMKIIα, *Shank3*

## Abstract

Single neurons, as the basic unit of the brain, consist of a cell body and processes, including dendrites and axons. Even neurons of the same type show various subtle process characteristics to fit into the diverse neural circuits. Different cell types of neurons form complicated circuits in the brain. Therefore, detailed neuronal morphology is required to understand normal neuronal function and pathological mechanisms, such as those that occur in autism. Here, we developed a strategy to sparsely label the same type of neurons throughout the whole brain and tested its application in an autistic animal model—*Shank3* knockout (KO) mice. To achieve this, we designed an adeno-associated virus (AAV) that expresses Cre recombinase-dependent regular and membrane-targeted enhanced green fluorescent protein (EGFP) under a human synapsin 1 promoter and verified it in several Cre transgenic mice. We could sparsely label the projection neurons in multiple brain areas by retro-ocular injection of the virus into CaMKIIα-Cre mice. Then, we analyzed the morphology of the projection neurons in *Shank3* KO mice with this method. We found differential dendritic complexity and dendritic spine changes in projection neurons in *Shank3* KO mice crossed with CaMKIIα-Cre mice compared with littermate control mice in the striatum, cortex, and hippocampus. By combining this method with various Cre mouse lines crossed with mouse models of disease, we can screen the morphological traits of distinct types of neurons throughout the whole brain that will help us to understand the exact role of the specific cell types of neurons not only in autism spectrum disorder (ASD) mouse models but also in other psychiatric disorder mouse models.

## Introduction

Neurons are the basic functional units of the nervous system. Billions of neurons form at least a thousand neuron types and integrate into various anatomical circuits for executing complex behaviors. In general, neurons are composed of cell bodies and processes, including dendrites and axons. These structures, particularly dendrites and dendritic spines, are the main subtle structures receiving inputs from neurons and glia cells, which display dynamic remodeling during development, aging, and diseases. Autism spectrum disorders (ASDs), one type of neurodevelopmental disease, have been found to induce morphological changes in the dendritic processes and spines of neurons in related brain regions (Penzes et al., 2011). Various types of neurons play distinct roles in different brain circuits that conduct normal brain functions. Although morphological changes in neurons have been revealed in ASD animal models (Bourgeron, 2009, 2015), structural deficits in different types of neurons have not been fully disclosed. In recent studies, growing evidence has shown that specific neuronal cell types contribute to the mechanisms of behavioral changes in ASD animal models (Tyzio et al., 2014; Bariselli et al., 2016; Zhang Y. et al., 2016; Wang et al., 2017; Guo et al., 2019). Moreover, a considerable number of studies have suggested an association between ASDs and dendritic spine abnormalities involving different stages, such as spine development, maturation, elimination, and pruning (Tang et al., 2014; Yadav et al., 2017; Yoon et al., 2020). Therefore, it is essential to characterize the morphological changes of distinct cell types in the brain circuits of those animal models of disease, which will greatly help us to understand the etiology of ASD, especially dendritic processes and dendritic spines. In addition, there are always a multitude of similar and different types of neurons that intermingle with each other, even in an anatomical region. Thus, single-cell reconstructions with sparse-labeling techniques are very useful for the scrutinization of the process traits of neurons, which will further help us understand the functional role of these neurons.

Many methods have been used to sparsely label single neurons in animal models. For instance, Peça et al. (2011) used the patch-assisted Lucifer yellow cell filling method to sparsely label neurons in the striatum, and they found that young *Shank3B* knockout (KO) mice, an ASD mouse model (we refer to *Shank3* KO throughout the paper), showed increased dendritic complexity and decreased dendritic spine density of medial spiny neurons (MSNs) compared with wild-type (WT) mice. Though this method can help us visualize the structure of single neurons in the brain, the efficiency is relatively low, and information on cell-type specificity is lacking. Zhang Q. et al. (2016) developed a strategy of exiguous labeling of neurons by using an adeno-associated virus (AAV) to express farnesylated enhanced green fluorescent protein (EGFPf). Recently, we confirmed that dendrite processes and dendritic spines showed clear deficits in the indirect-pathway MSNs of the striatum and pyramidal cells in the anterior cingulate cortex in *Shank3* KO mice by using this strategy (Wang et al., 2017; Guo et al., 2019). However, it is time-consuming to achieve cell-type-specific labeling using this method. We had to combine this method with a transgenic reporter mouse line to distinguish striatonigral MSNs from striatopallidal MSNs. Therefore, a more efficient and simpler way to target a defined type of neurons will be useful for us to study the neuronal morphological changes in ASD models.

In this study, we modified an AAV expressing EGFP and EGFPf (Zhang Q. et al., 2016) into a Cre recombinase-dependent version. We performed retro-orbital injection of this new virus into CaMKIIα-Cre transgenic mice with different titers and successfully achieved single-cell-type-specific labeling in the different brain regions. The expressions of both EGFP and EGFPf enabled us to visualize the detailed structures of each neuron, even the spines. To explore the cell-type-specific neuronal morphological changes of ASD animal models, we applied this new method in *Shank3* KO mice, which were crossed with CaMKIIα-Cre transgenic mice. We compared dendritic complexity and dendritic spines of the labeled neurons between WT and *Shank3* KO mice in different brain regions. Our results showed that dendritic complexity and spine density were reduced to various degrees in the striatum, cortex, and dentate gyrus (DG) of this ASD mouse model. This highly efficient single-cell-type-specific labeling method allowed us to visualize the single-cell morphology not only in normal brains but also in autistic brains.

## Materials and Methods

### Vector Preparation

To make the pAAV-hSyn-DIO-EGFP-P2A-EGFPf construct, EGFP and EGFPf sequences were PCR amplified from a pAAV-hSyn-EGFP-P2A-EGFPf-WPRE-HGHpA construct (Addgene, #74513). The loxP, loxP2272, and P2A sequences were added during the PCR amplification. The primer sequences were as follows: DIO-GFPf-F1: 5′-ATTGTAGCTGCTATT AGCAATATGAAACCTCTTAATAACTTCGTATAGCATACAT TATACGAAGTTATTCAGGAGAGCACACACTTGC-3′, DIO-GFPf-R1: 5′-GAATAACAGTGATAATTTCTGGGTTAAGGCA AATAACTTCGTATAGGATACTTTATACGAAGTTATGCCAC CATGGTGAGCAAGGGCG-3′, DIO-GFPf-F2: 5′-ACCGG CTAGAGGATCCATAACTTCGTATAGGATACTTTATACGAA GTTATGCAGAATGGTAGCTGGATTGTAGCTGCTATTAGC AA-3′, and DIO-GFPf-R2: 5′-GATTATCGATAAGCTTATAAC TTCGTATAGCATACATTATACGAAGTTATTCTTTGCACCA TTCTAAAGAATAACAGTGATAATTTCTGG-3′.

PCR-amplified fragments were ligated with *Bam*HI and *Hin*dIII linearized pAAV-hSyn-EGFP-P2A-EGFPf-WPRE-HGHpA construct using an Infusion cloning kit (Takara Bio Inc., Japan). The pAAV-hSyn-DIO-EGFP-P2A-EGFPf construct was confirmed with Sanger sequencing.

### Mice

All procedures were approved by the Institutional Animal Care and Use Committee of the Fourth Military Medical University (FMMU). Mice were housed in a room maintained at a constant temperature and on a 12-h light/dark cycle (light from 08:00 to 20:00). Water and food were available *ad libitum*. *Shank3B* KO mice were gifts from Guoping Feng’s laboratory at MIT. The CaMKIIα-Cre transgenic line was from the Jackson Laboratory (calcium/calmodulin-dependent protein kinase II alpha promoter driving Cre recombinase expression, stock no.: 005359), and *Sst*-IRES-Cre knock-in mice were from the Jackson Laboratory (express Cre recombinase in somatostatin-expressing neurons, stock no.: 013044). *Shank3*^+/+^:CaMKIIα-Cre hemizygotes and *Shank3*^–/–^:CaMKIIα-Cre hemizygotes were obtained from *Shank3*^+/–^:CaMKIIα-Cre hemizygotes crossed with *Shank3*^+/–^ mice. Ten-week-old male and female mice were handled by experimenters who were blinded to the genotypes and groups.

### Cell Culture

Primary dissociated cortical neurons were prepared from postnatal day 0 WT mice by using standard protocols, as previously described (Ramamoorthi et al., 2011). Cortical neurons (2 × 10^5^) were plated into one well of a 24-well plate that was precoated with 20 μg ml^–1^ poly-D-lysine (Sigma–Aldrich, United States) and 4 μg ml^–1^ laminin (Life Technologies, United States). The cultures were treated with AraC (1 μg ml^–1^; Sigma–Aldrich, United States) on day 5 *in vitro* and maintained for up to 18 days after plating. One microliter of AAV_*PhP.eB*_-hSyn-DIO-EGFP-P2A-EGFPf 1.3E + 12 gc/ml with 1 μl of AAV_8_-CaMKIIα-ΔCre-mKate2 (1.0E + 13 gc/ml) or AAV_8_-CaMKIIα-Cre-mKate2 (1.0E + 13 gc/ml) was added to one well at DIV10, and neurons were fixed with 4% paraformaldehyde (PFA) for imaging at DIV18. With these viral titers, almost all pyramidal neurons were infected by AAV_8_-CaMKIIα-ΔCre-mKate2 or AAV_8_-CaMKIIα-Cre-mKate2, which expressed red fluorescence. We observed that ∼80% of pyramidal neurons expressed EGFP and red fluorescence when infected with both AAV_8_-CaMKIIα-Cre-mKate2 and AAV_*PhP.eB*_-hSyn-DIO-EGFP-P2A-EGFPf virus.

### AAV Production and Purification

The AAV was packaged by PackGene Biotech in China. Triple-plasmid transfection using polyethylenimine (PEI MAX, Cat. 24765; Polysciences, United States) was carried out to produce the recombinant AAV. The plasmids pAAV-hSyn-DIO-EGFP-P2A-EGFPf, pHelper, and pRep2Cap-PhP.eB, which encode the Rep2 and PhP.eB capsid proteins, were cotransfected into HEK293T cells. The cells were cultured in Dulbecco’s modified essential medium (DMEM; Invitrogen, United States), containing 10% fetal bovine serum (Gibco, United States) and 1% penicillin–streptomycin, in an incubator at 37°C with 5% CO_2_. HEK293T cells were seeded in 150-mm dishes at a density of 1 × 10^7^ cells per dish 24 h prior to transfection. Cells were transfected with 12 μg of pHelper plasmid, 10 μg of AAV pRep2Cap-PhP.eB plasmid, and 6 μg of pAAV-hSyn-DIO-EGFP-P2A-EGFPf plasmid for each plate. At 72 h post transfection, cells were harvested by 4000 *g* centrifugation at 4°C for 30 min. The pellet was collected and resuspended in buffer containing 10 mM Tris-HCl, pH 8.0. The suspension was subjected to four freeze–thaw cycles by dry ice/ethanol and a 37°C water bath. The cell debris was sonicated and then digested with DNase I (200 units in 1.5 ml) for 1 h at 37°C. Following centrifugation at 10,000 *g* for 10 min at 4°C, the supernatant was collected as the AAV crude lysate. The crude lysate was diluted with 10 mM Tris-HCl pH 8.0 to a final volume of 10 ml and then bottom-loaded on a discontinuous gradient of 15, 25, 40, and 60% iodixanol in a 39-ml ultracentrifuge tube (QuickSeal, 342414, Beckman, United States). After ultracentrifugation at 350,000 *g* at 18°C for 1 h, 3-ml fractions in the 40% lower layer and 0.5 ml of the 60% upper layer were collected. The fractions were desalted using a 100-kDa cutoff ultrafiltration tube (15 ml; Millipore, United States), and the buffer was changed to phosphate buffer saline (PBS). The purified AAV was stored at −80°C until use. The AAV genome copy titers were determined by real-time quantitative PCR (qPCR).

### Retro-Ocular Injection

Mice were given retro-ocular injection of AAV as previously described (Yardeni et al., 2011; Zhang Q. et al., 2016). Briefly, mice were anesthetized with 3% isoflurane (RWD Life Science, China). Then, 100 μl of AAV_*PhP.eB*_-DIO-EGFP-P2A-EGFPf with a titer of 1.3E + 12 gc/ml (PackGene Biotech, LLC, China) was injected into the retro-orbital sinus with a 27-G needle and a 1-ml syringe. The mice were then placed in a warm and moist environment to wait for resuscitation. After resuscitation, the mice were put back in the cage and housed for 3 weeks before the next step.

### Immunohistochemistry

Mice were anesthetized with sodium pentobarbital (1%, 40 mg/kg body weight, i.p.) and transcardially perfused with 0.01 M PBS followed by 4% PFA in 0.01 M PBS. Brains were dissected out and postfixed in the same fixative at 4°C overnight. After that, brains were sliced in 200-μm-thick coronal sections by using a Vibratome 1000 (Ted Pella Inc., United States). All sections were serially collected into light-protected six-well plates containing 0.01 M PBS and were stored at 4°C for subsequent immunohistochemical staining.

Brain slices were rinsed once with PBS and permeated with 0.5% Triton X-100 (234729, Sigma, United States) in PBS for 2 h at room temperature. Then, the slices were rinsed once with PBS and incubated with 15% normal goat serum, 5% BSA, and 0.2% Triton X-100 (GBT) for 1 h at room temperature. After that, slices were incubated with primary antibody (Supplementary Table S1) at 4°C for 48 h. Then, the slices were washed with GBT five times at room temperature, each time for 15 min. Slices were incubated at 4°C for 12 h with the secondary antibody (Supplementary Table S1) and then stained with DAPI (1:1000, D8417, Sigma, United States) in PBS for 15 min at room temperature. Slices were washed five times in PBS with 0.1% Tween-20 and one time in PBS at room temperature, each time for 15 min. All of the above operations were performed on a shaking table with stirring (100 rpm/min). Last, brain slices were mounted on slides with a Fluoromount-G mounting medium (0100-01, Southern Biotech, United States).

### Neuronal Imaging and Analysis

An Olympus FV1000 confocal microscope and Olympus FLUOVIEW software (ver.1.7a, Olympus, Japan) were used for image acquisition. Images were acquired at a resolution of 1024 pixels in the *X*–*Y* dimension. *Z* dimensions were variable. To obtain the overall distribution of EGFP-labeled neurons throughout the whole brain, a low-power objective lens (10×, numerical aperture = 0.4) was used to obtain the images, and then XuvStitch software, Ver. 1.8.099 (Emmenlauer et al., 2009), was used to stitch the images. The number of EGFP-labeled neurons in similar brain regions of WT and KO mice was counted manually. For the analysis of dendritic branches, most of the neurons were imaged using a 40× objective lens (numerical aperture = 0.9). Since the pyramidal neurons in layers V–VI of the cortex are larger than those in the other regions, a 20× objective lens (numerical aperture = 0.9) was used for imaging. The *Z*-dimensional increment was 1 μm for *Z*-stack images in both conditions. A 60× objective lens (numerical aperture = 1.42) was used for dendritic spine imaging. The *Z*-dimensional increment was 0.1–0.3 μm for the spine *Z*-stack images.

Neurons selected for analysis were randomly picked from at least 10 brain slices of three WT and three *Shank3* KO mice. The stack images were analyzed using Imaris software (version 7.7.1, serial number: 32mr-rfhf-7hbu-jb58, Bitplane, Switzerland). To eliminate the interference of neighboring neuronal dendrites, the confocal imaging files were converted to Imaris file format with the “Imaris File Converter,” and 3D reconstruction of a single neuron was performed using the “volume rendering” function. The pictures shown in this paper were captured using the “snapshot” tools. The total length of dendrites and the volume of the cell body were automatically calculated. For the Sholl analysis, the spheres were constructed continuously from the center of the cell body with the radius increased by 10 μm. The number of intersections between each sphere and dendrites was calculated for comparison. Compared with using 2D images, using 3D images for Sholl analysis can provide statistical results that are closer to the real structure of the neurons, especially when analyzing dendrites with different angles.

To compare the difference in spine density between the WT and KO mice, spines in secondary dendrites with 80-μm length were chosen for the analysis. The spines in the dendrites were detected by Imaris software semiautomatically. The mislabeled spines were deleted manually. Spine shape was defined by the length of the spine and the width of the spine neck and the spine head, which allowed us to classify the spines into four types: stubby, mushroom, long thin, and filopodia. The stubby type had a length < 1 μm; the mushroom type had a length > 3 μm, and the maximum width of the head/the mean of the neck was > 2; the long thin type had a ratio of the mean width of the head/mean width of the neck of ≥1; and the rest of the spines were filopodia. Spine measurements were performed using Matlab-XTension Spines Classifier in Imaris. All imaging data were analyzed by a person who was blinded to the experimental groups.

### Statistical Analysis

All data were transferred to SPSS 21.0 (IBM, United States) for analysis and to Prism 6.0 (GraphPad Software, Inc., United States) for graphing. All data were subjected to the normality test by the Shapiro–Wilk test and the homogeneity of variance test by Levene’s test before further statistical analysis. Data are presented as the mean ± SEM, and the *n* value given for each experiment refers to the number of cells analyzed. All error bars indicate the SEM. Details of the particular statistical methods and results can be found in Supplementary Tables S2, S3. The results of two-group comparisons were analyzed by using a two-tailed unpaired *t*-test except as mentioned in the text. The significance levels for all tests were set at ^∗^*p* < 0.05, ^∗∗^*p* < 0.01, ^∗∗∗^*p* < 0.001, and ^****^p < 0.0001.

## Results

### Construction of the AAV-hSyn-DIO-EGFP-P2A-EGFPf Vector

**FIGURE 1:**
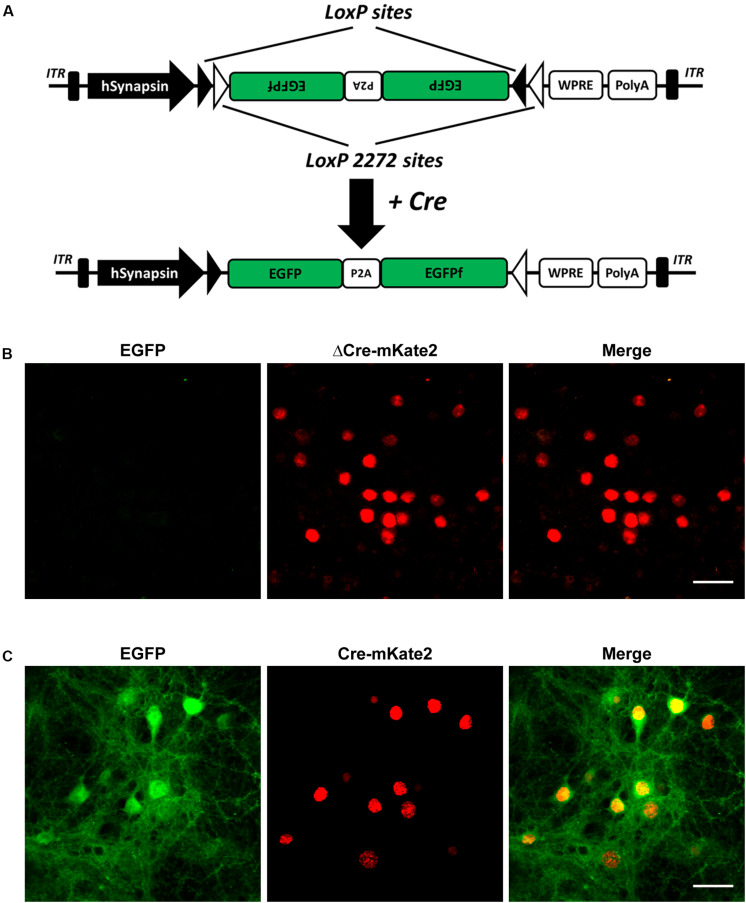
Selective CaMKIIα-Cre virus mediated EGFP expression of AAV-hSyn-DIO-EGFP-P2A-EGFPf in primary cultured cortical neurons. **(A)** Schematic illustration of the AAV-hSyn-DIO-EGFP-P2A-EGFPf construct. Expression of EGFP under the control of the neuron-specific human *synapsin* promoter (hSyn). EGFP and EGFPf are floxed by a pair of loxP (filled triangles) and lox2272 (empty triangles) sites. In the absence of Cre recombinase, the EGFP and EGFPf coding sequences are inverted relative to the hSyn promoter. When expressed, Cre recombinase inverts the EGFP and EGFPf sequences into an active orientation by “flipping” and then “locking” the lox2272 and loxP sites. ITR, inverted terminal repeat; P2A, porcine teschovirus-1 2A; WPRE, Woodchuck hepatitis virus posttranscriptional regulatory element. **(B)** Confocal images of primary cultured cortical neurons infected with AAV_8_-CaMKIIα−ΔCre-mKate2 and AAV_*PhP.eB*_-Syn-DIO-EGFP-P2A-EGFPf virus (negative control). **(C)** Confocal images of primary cultured cortical neurons infected with AAV_8_-CaMKIIα-Cre-mKate2 and AAV_*PhP.eB*_-hSyn-DIO-EGFP-P2A-EGFPf. Images show EGFP (green) and ΔCre/Cre-mKate2 (red) expression. Scale bar: 50 μm.

To achieve neuronal cell-type-specific labeling *in vivo*, we chose the double-floxed inversed orientation (DIO) switch system, also known as the flip-excision (FLEX) Cre-switch system, to control EGFP reporter expression with Cre recombinase driven by specific promoters (Atasoy et al., 2008). The FLEX Cre-switch system consists of two pairs of heterotypic loxP-variant recombination sites, namely, loxP, having the WT sequence, and lox2272, having a mutated sequence flanking a pair of open reading frames. Both loxP variants are recognized by Cre recombinase, but only identical pairs of loxP sites can recombine with each other and not with any other variants (Figure 1A). A previous study showed that the detailed neuronal structure, including the dendrites and spines, can be visualized with an AAV-expressing EGFP and membrane-targeted EGFPf linked by self-cleaving P2A peptide (EGFP-P2A-EGFPf, hereafter referred to as EGFPf) (Zhang Q. et al., 2016). Encouraged by this, we inserted the inversed EGFPf coding sequence floxed by a pair of loxP and lox2272 sites into an AAV construct with the human *synapsin* promoter, which drives gene expression in neurons. In the absence of Cre recombinase, EGFPf is not expressed due to its antisense orientation. In the presence of Cre recombinase, the loxP and lox2272 sites undergo recombination with the other loxP and lox2272 sites, respectively, resulting in the inversion of EGFPf and the excision of one from each pair of identical recombination sites. Then, EGFPf is expressed in a Cre-dependent manner (Figure 1A). To verify whether this strategy works and how stringent it is, we tested this system in primary cultured neurons first. We transfected AAV_8_-CaMKIIα-ΔCre-mKate2 and AAV_*PhP.eB*_-Syn-DIO-EGFP-P2A-EGFPf virus into primary cultured cortical neurons as a negative control. In ΔCre, the catalytic domain of Cre recombinase was deleted. The neurons infected with these two viruses showed only a red color, without the expression of EGFPf, which suggested that the expression of ΔCre-mKate2 was not able to turn on the expression of EGFPf (Figure 1B). We infected primary cultured cortical neurons with AAV_8_-CaMKIIα-Cre-mKate2 and AAV_*PhP.eB*_-DIO-EGFP-P2A-EGFPf virus. Strong EGFPf expression was visualized in red AAV_8_-CaMKIIα-Cre-mKate2-infected neurons (Figure 1C). From these data, we confirmed that the expression of EGFPf in our construct depended on Cre recombinase driven by a specific promoter.

### Sparse Labeling of CaMKIIα-Positive Neurons Throughout the Whole Brain

**FIGURE 2:**
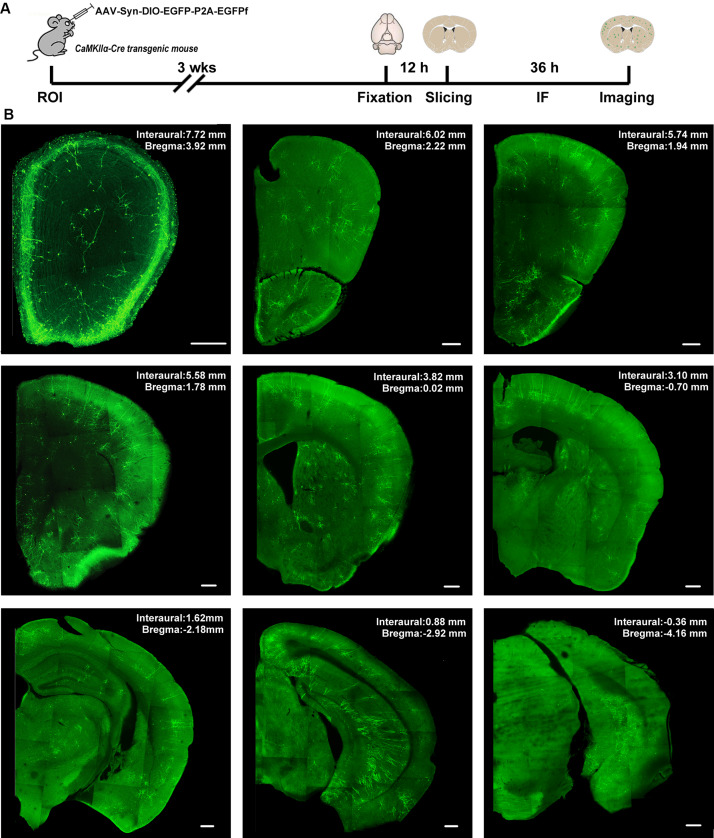
The expression of AAV-hSyn-DIO-EGFP-P2A-EGFPf in the CaMKIIα-Cre transgenic mouse brain. **(A)** Schematic illustration of the experimental workflow. ROI, retro-orbital injection; IF, immunofluorescence staining. **(B)** A series of confocal images shows the distribution of sparsely labeled neurons in different brain regions from a CaMKIIα-Cre transgenic mouse. Scale bar: 300 μm.

Systematic AAV injection provides an alternative non-invasive method for the broad delivery of genes to the nervous system. To achieve cell-type-specific labeling of neurons throughout the whole brain, we performed retro-orbital injection of AAV_*PhP.eB*_-hSyn-DIO-EGFP-P2A-EGFPf virus in CaMKIIα-Cre transgenic mice (Figure 2A). AAV-PhP.eB is a novel capsid that can transduce the majority of neurons across many regions of the adult mouse brain and spinal cord after intravenous injection (Chan et al., 2017). We titrated the injected viral load to achieve sparse labeling of the neurons in the adult mouse brain. To visualize all labeled neurons, we performed immunofluorescence histochemistry with an EGFP antibody to amplify the EGFPf signal. A series of images in Figure 2B show a CaMKIIα-Cre mouse brain, from rostral to caudal, in the low-magnification field 3 weeks after viral injection. The morphology of all labeled neurons could be easily identified in those sections.

**FIGURE 3:**
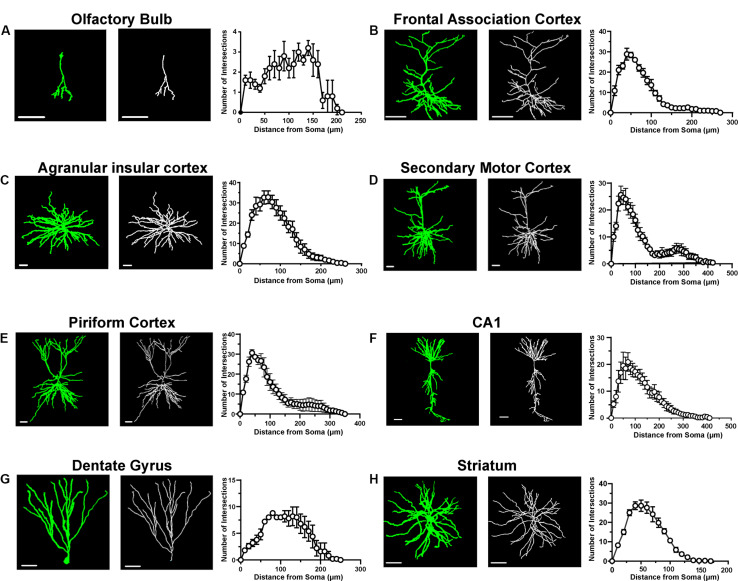
Morphological analysis of single neurons in different brain areas. **(A)** A representative labeled neuron and its reconstructed image (left panel) in the olfactory bulb from a CaMKIIα-Cre transgenic mouse brain; Sholl analysis of labeled neurons in the olfactory bulb (right panel, *n* = 5 neurons from three mice). **(B)** A representative labeled neuron and its reconstructed image (left panel) in the frontal association cortex from a CaMKIIα-Cre transgenic mouse brain; Sholl analysis of labeled neurons in the frontal association cortex (right panel, *n* = 5 neurons from three mice). **(C)** A representative labeled neuron and its reconstructed image (left panel) in the agranular insular cortex from a CaMKIIα-Cre transgenic mouse brain; Sholl analysis of labeled neurons in the agranular insular cortex (right panel, *n* = 5 neurons from three mice). **(D)** A representative labeled neuron and its reconstructed image (left panel) in the secondary motor cortex from a CaMKIIα-Cre transgenic mouse brain; Sholl analysis of labeled neurons in the secondary motor cortex (right panel, *n* = 5 neurons from three mice). **(E)** A representative labeled neuron and its reconstructed image (left panel) in the piriform cortex from a CaMKIIα-Cre transgenic mouse brain; Sholl analysis of labeled neurons in the piriform cortex (right panel, *n* = 5 neurons from three mice). **(F)** A representative labeled neuron and its reconstructed image (left panel) in the CA1 of the hippocampus from a CaMKIIα-Cre transgenic mouse brain; Sholl analysis of labeled neurons in the CA1 of the hippocampus (right panel, *n* = 5 neurons from three mice). **(G)** A representative labeled neuron and its reconstructed image (left panel) in the dentate gyrus from a CaMKIIα-Cre transgenic mouse brain; Sholl analysis of labeled neurons in the dentate gyrus (right panel, *n* = 5 neurons from three mice). **(H)** A representative labeled neuron and its reconstructed image (left panel) in the striatum from a CaMKIIα-Cre transgenic mouse brain; Sholl analysis of labeled neurons in the striatum (right panel, *n* = 5 neurons from three mice). Scale bar: 50 μm.

To assess the specificity of this strategy, we performed immunofluorescent staining with an anti-CaMKIIα antibody to test whether the EGFPf-labeled neurons were CaMKIIα-positive neurons. As expected, EGFPf fluorescence colocalized with CaMKIIα immunostaining in various regions, including the cortex and striatum, which are the major expression areas of the endogenous CaMKIIα protein (Supplementary Figure S1). Interestingly, we found that some EGFPf-labeled neurons were CaMKIIα positive in the hilus region of the DG, in which there are many GABAergic interneurons. Next, we performed 3D reconstructions of EGFPf-labeled neurons in different brain regions and analyzed the complexity of their dendritic trees. In Figure 3 and Supplementary Figure S2, we exhibit some examples of the original images and their 3D reconstructed images of EGFPf-labeled neurons from CaMKIIα-Cre mice. EGFPf-labeled neurons presented a classic pyramidal shape in the frontal association cortex, secondary motor cortex, piriform cortex, and CA1 in the hippocampus. However, the detailed dendritic processes of these neurons were conspicuously divergent. In addition, some EGFPf-labeled neurons also showed different shapes in other areas. For example, in the striatum, the EGFPf labeling depicted the radial dendritic pattern that fits in the projection GABAergic medium-sized spiny neurons (MSNs). In the DG and olfactory bulb, the green fluorescence indicated the granule cell shape. In the agranular insular cortex, some EGFPf even labeled non-pyramidal cell types (Figure 3 and Supplementary Figure S2). Our results were similar to a previous finding using CaMKIIα-GFP mice (Wang et al., 2013).

To broaden the application of our strategy in other Cre transgenic mouse lines, we tested the labeling effect with a *Somatostatin* Cre transgenic mouse line (SST-Cre) in which Cre is expressed in SST-positive interneurons in the brain. After viral injection, we found that EGFPf-labeled neurons showed morphological traits of interneurons (Supplementary Figure S3). The EGFPf-labeled neurons were SST protein-positive interneurons as revealed by SST immunostaining (Supplementary Figure S3). These results further confirmed that our strategy was suitable for cell-type-specific labeling and could potentially be applied in other Cre transgenic mouse lines.

### Visualization of the Morphological Traits of CaMKIIα-Positive Neurons in *Shank3* KO Mouse Brains

**FIGURE 4:**
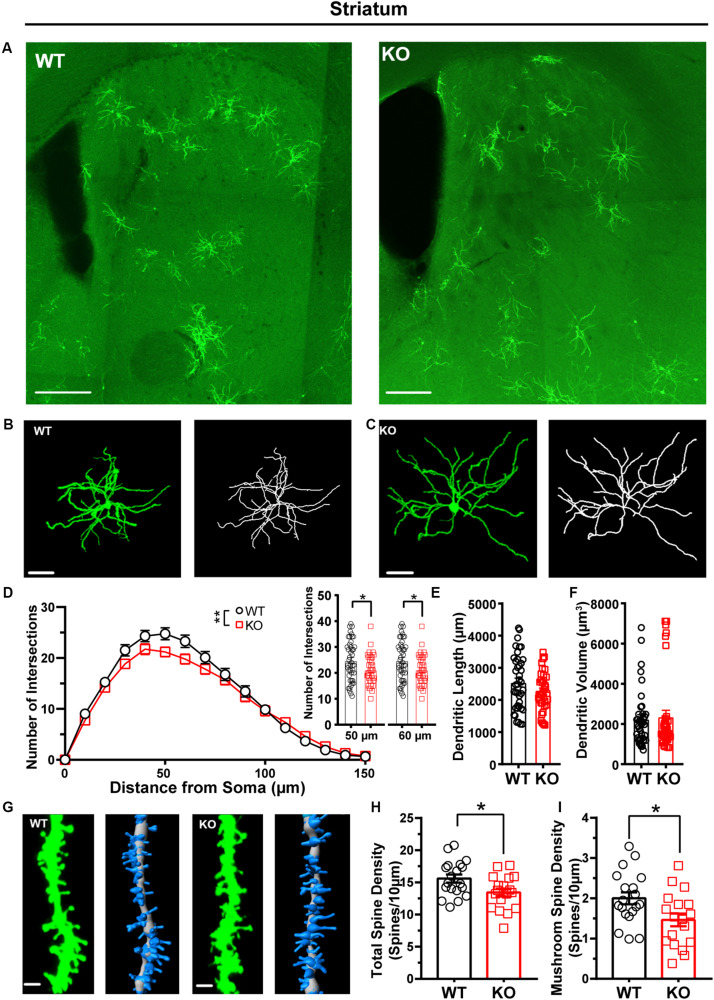
Dendritic complexity and dendritic spine density were reduced in medium-sized spiny neurons (MSNs) from the striatum of *Shank3* KO mice. **(A)** Representative images showing the distribution of labeled neurons in the striatum of WT and KO mice. Scale bar: 200 μm. Representative projection neurons and reconstructed images in the striatum of WT **(B)** and KO mice **(C)**. Scale bar: 50 μm. **(D)** Sholl analysis showed reduced dendritic complexity of MSNs in KO mice compared with WT mice, and specific differences existed between WT and KO mice in Sholl radius (WT: *n* = 44 neurons from three mice; KO: *n* = 37 neurons from three mice, Friedman’s *M* test, χ^2^ = 7.750, *df* = 1, *p* = 0.005; Sholl radius in 50 μm, WT: 24.77 ± 1.19, KO: 21.19 ± 0.94, two-tailed unpaired *t*-test, *t* = 2.30, *df* = 79, *p* = 0.02; Sholl radius in 60 μm, WT: 23.3 ± 1.24, KO: 19.78 ± 0.98, two-tailed unpaired *t*-test, *t* = 2.17, *df* = 79, *p* = 0.03). **(E)** The dendritic length of MSNs was similar between KO mice and WT mice (WT: 2,544 ± 135.5 μm, *n* = 40 neurons from three mice; KO: 2272 ± 99.64 μm, *n* = 36 neurons from three mice, two-tailed unpaired separate variance estimation *t*-test, *t* = 1.62, df = 69.84, *p* = 0.11). **(F)** The dendritic volume of MSNs was similar between KO mice and WT mice (WT: 2246 ± 217.7 μm^3^, *n* = 40 neurons from three mice; KO: 2356 ± 323.2 μm^3^, *n* = 37 neurons from three mice, Mann–Whitney *U*-test, *Z* = -0.91, *p* = 0.36). **(G)** Representative spine and reconstructed images in the striatum of WT (left panel) and KO mice (right panel). Scale bar: 2 μm. **(H)** The total dendritic spine density was reduced in KO mice compared with WT mice (WT: 15.58 ± 0.63/10 μm, *n* = 19 dendrites from three mice; KO: 13.43 ± 0.62/10 μm, *n* = 18 dendrites from three mice, two-tailed unpaired *t*-test, *t* = 2.44, *df* = 35, *p* = 0.02). **(I)** The density of mushroom spines was reduced in KO mice compared with WT mice (WT: 2.00 ± 0.15/10 μm, *n* = 19 dendrites from three mice; KO: 1.46 ± 0.16/10 μm, *n* = 18 dendrites from three mice; two-tailed unpaired *t*-test, *t* = 2.47, *df* = 35, *p* = 0.02). Data are presented as the mean ± SEM. ^∗^*p* < 0.05, ^∗∗^*p* < 0.01. WT, wild-type mice; KO, *Shank3* KO mice.

After we confirmed that the FLEX-EGFPf switch system can successfully label single neurons in CaMKIIα-Cre transgenic mice, we wanted to test whether this method could also be used to visualize the morphological traits of single neurons in *Shank3* KO autistic mouse models. We crossed *Shank3* heterozygous mice with CaMKIIα-Cre transgenic mice to obtain *Shank3* KO:CaMKIIα-Cre mice and their littermate controls (WT:CaMKIIα-Cre, Supplementary Figure S4). After that, we performed retro-orbital injection of AAV_*PhP.eB*_-hSyn-DIO-EGFP-P2A-EGFPf virus in these mice and analyzed the labeled neurons in several brain areas. Previous studies have shown that the striatum is the major impaired brain region in *Shank3* KO mice (Peça et al., 2011; Mei et al., 2016; Zhou et al., 2016; Wang et al., 2017). First, we performed 3D reconstruction of MSNs and analyzed the neuronal morphological traits of WT and *Shank3* KO mice in the striatum (Figures 4A–C). The results showed that the dendritic complexity in *Shank3* KO mice was reduced compared with that in WT mice (Figure 4D, WT: *n* = 44 neurons from three mice; KO: *n* = 37 neurons from three mice, Friedman’s *M* test, *p* = 0.005). And we found reduced intersections in 50 and 60 μm *Shank3* KO mice compared with WT mice (Sholl radius in 50 μm, WT: 24.77 ± 1.19, KO: 21.19 ± 0.94, *p* = 0.02; Sholl radius in 60 μm, WT: 23.3 ± 1.24, KO: 19.78 ± 0.98, *p* = 0.03). There was no difference in dendritic length or volume between *Shank3* KO and WT mice (Figures 4E,F, dendritic length, WT: 2,544 ± 135.5 μm, *n* = 40 neurons from three mice, KO: 2,272 ± 99.64 μm, *n* = 36 neurons from three mice, two-tailed unpaired separate variance estimation *t*-test, *p* = 0.11; dendritic volume, WT: 2,246 ± 217.7 μm^3^, *n* = 40 neurons from three mice, KO: 2,356 ± 323.2 μm^3^, *n* = 37 neurons from three mice, Mann–Whitney *U*-test, *p* = 0.36). The total dendritic spine density was reduced in *Shank3* KO mice compared with WT mice (Figures 4G,H, total spine density, WT: 15.58 ± 0.63/10 μm, *n* = 19 dendrites from three mice, KO: 13.43 ± 0.62/10 μm, *n* = 18 dendrites from three mice, *p* = 0.02). Among the different spine types, the density of mushroom and long thin spines reduced, but the density of the stubby and filopodia spines did not (Figures 4G,I and Supplementary Figures S6A–C, mushroom spine density, WT: 2.0 ± 0.15/10 μm, *n* = 19 dendrites from three mice, KO: 1.46 ± 0.16/10 μm, *n* = 18 dendrites from three mice, *p* = 0.02; stubby spine density, WT: 2.37 ± 0.23/10 μm, *n* = 19 dendrites from three mice, KO: 2.65 ± 0.33/10 μm, *n* = 18 dendrites from three mice, *p* = 0.49; long thin spine density, WT: 1.30 ± 0.19/10 μm, *n* = 19 dendrites from three mice, KO: 0.72 ± 0.15/10 μm, *n* = 18 dendrites from three mice, *p* = 0.03; filopodia spine density, WT: 9.91 ± 0.66/10 μm, *n* = 19 dendrites from three mice, KO: 8.59 ± 0.43/10 μm, *n* = 18 dendrites from three mice, two-tailed separate variance estimation, *p* = 0.10).

**FIGURE 5:**
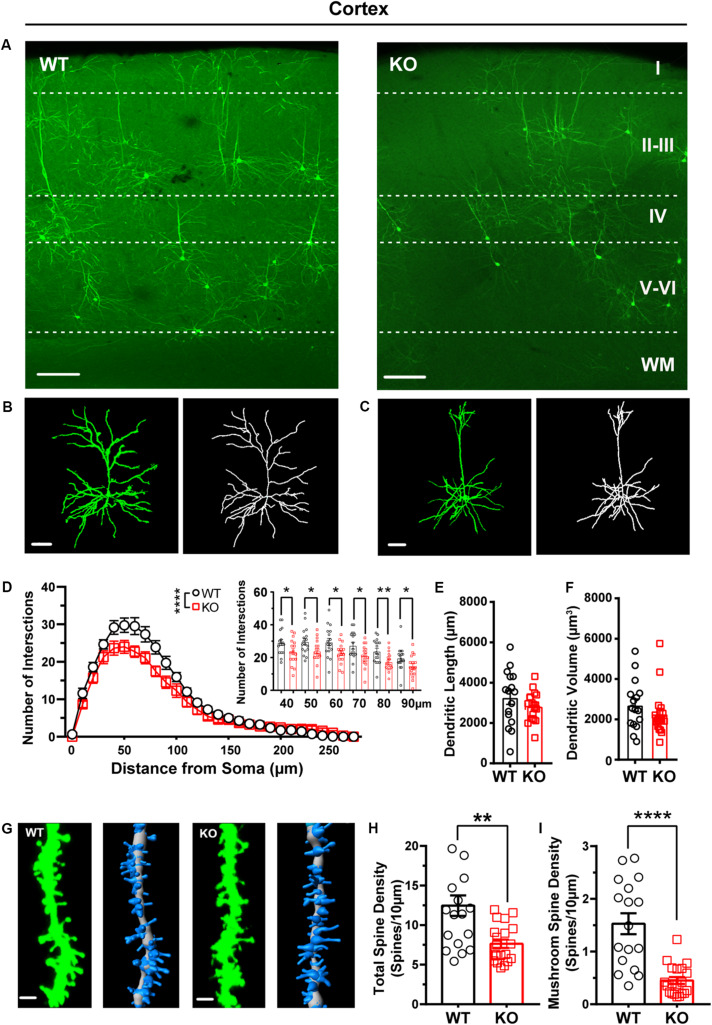
Dendritic complexity and dendritic spine density were reduced in pyramidal neurons from the cortex of *Shank3* KO mice. **(A)** Representative images showing the distribution of labeled neurons in the cortex of WT and KO mice. Scale bar: 200 μm. WM: white matter. Representative projection neurons and reconstructed images in the cortex of WT **(B)** and KO mice **(C)**. Scale bar: 50 μm. **(D)** Sholl analysis showed reduced dendritic complexity of pyramidal neurons in KO mice compared with WT mice, and specific differences existed between WT and KO mice in Sholl radius (WT: *n* = 17 neurons from three mice, KO: *n* = 17 neurons from three mice, Friedman’s *M* test, χ^2^ = 12.63, *df* = 1, *p* < 0.0001; Sholl radius in 40 μm, WT: 29.18 ± 1.82, KO: 23.65 ± 1.87, two-tailed unpaired *t*-test, *t* = 2.12, *df* = 32, *p* = 0.04; Sholl radius in 50 μm, WT: 29.76 ± 1.93, KO: 24 ± 1.71, two-tailed unpaired *t*-test, *t* = 2.24, *df* = 32, *p* = 0.03; Sholl radius in 60 μm, WT: 29.53 ± 2.24, KO: 22.71 ± 1.50, two-tailed unpaired *t*-test, *t* = 2.53, *df* = 32, *p* = 0.02; Sholl radius in 70 μm, WT: 27.35 ± 2.04, KO: 21.06 ± 1.59, two-tailed unpaired *t*-test, *t* = 2.44, *df* = 32, *p* = 0.02; Sholl radius in 80 μm, WT: 23.88 ± 1.78, KO: 17.47 ± 1.38, two-tailed unpaired *t*-test, *t* = 2.84, *df* = 32, *p* = 0.008; Sholl radius in 90 μm, WT: 19.71 ± 1.73, KO: 14.53 ± 1.63, two-tailed unpaired *t*-test, *t* = 2.18, *df* = 32, *p* = 0.04). **(E)** The dendritic lengths of pyramidal neurons were similar between KO mice and WT mice (WT: 3,265 ± 343.4 μm, *n* = 16 neurons from three mice; KO: 2,831 ± 171 μm, *n* = 18 neurons from three mice, two-tailed unpaired separate variance, *t* = 1.13, *df* = 22.16, *p* = 0.27). **(F)** The dendritic volume of pyramidal neurons was similar between KO mice and WT mice (WT: 2705 ± 306.1 μm^3^, *n* = 16 neurons from three mice; KO: 2269 ± 266.8 μm^3^, *n* = 18 neurons from three mice. Mann–Whitney *U*-test, *Z* = -1.28, *p* = 0.20). **(G)** Representative spine and reconstructed images in the cortex of WT (left) and KO mice (right). Scale bar: 2 μm. **(H)** The total dendritic spine density was reduced in KO mice compared with WT mice (WT: 12.46 ± 1.29/10 μm, *n* = 18 dendrites from three mice; KO: 7.64 ± 0.53/10 μm, *n* = 20 dendrites from three mice; two-tailed unpaired separate variance estimation *t*-test, *t* = 3.46, *df* = 22.71, *p* = 0.002). **(I)** The density of mushrooms was reduced in KO mice compared with WT mice (WT: 1.5 ± 0.20/10 μm, *n* = 17 dendrites from three mice; KO: 0.45 ± 0.06/10 μm, *n* = 20 dendrites from three mice. Mann–Whitney *U*-test, *Z* = -4.09, *p* < 0.0001). Data are presented as the mean ± SEM. *p < 0.05, **p < 0.01, ****p < 0.0001. WT, wild-type mice; KO, *Shank3* KO mice.

Our recent work shows that the cortex exhibited neuronal morphological deficits in *Shank3* KO mice (Guo et al., 2019). Thus, we analyzed the dendritic processes and spines of pyramidal neurons in the cortex of WT and *Shank3* KO mice. The Sholl analysis results showed that the dendritic complexity of *Shank3* KO pyramidal neurons was reduced compared with that of WT mice (Figures 5A–D, WT: *n* = 17 neurons from three mice, KO: *n* = 17 neurons from three mice, Friedman’s *M* test, *p* < 0.0001). We found a reduction in intersections of 40–90 μm in *Shank3* KO mice compared to WT (Sholl radius in 40 μm, WT: 29.18 ± 1.82, KO: 23.65 ± 1.87, *p* = 0.04; Sholl radius in 50 μm, WT: 29.76 ± 1.93, KO: 24 ± 1.71, *p* = 0.03; Sholl radius in 60 μm, WT: 29.53 ± 2.24, KO: 22.71 ± 1.50, *p* = 0.02; the Sholl radius in 70 μm, WT: 27.35 ± 2.04, KO: 21.06 ± 1.59, *p* = 0.02; Sholl radius in 80 μm, WT: 23.88 ± 1.78, KO: 17.47 ± 1.38, *p* = 0.008; Sholl radius in 90 μm, WT: 19.71 ± 1.73, KO: 53 ± 1.63, *p* = 0.04). There was no significant reduction in the total dendritic length or volume of labeled neurons in *Shank3* KO mice compared with WT mice (Figures 5E,F, dendritic length, WT: 3265 ± 343.4 μm, *n* = 16 neurons from three mice, KO: 2831 ± 171.0 μm, *n* = 18 neurons from three mice, two-tailed unpaired separate variance, *p* = 0.27; dendritic volume, WT: 2705 ± 306.1 μm^3^, *n* = 16 neurons from three mice, KO: 2269 ± 266.8 μm^3^, *n* = 18 neurons from three mice, Mann–Whitney *U*-test, *p* = 0.20). The total dendritic spine density was reduced significantly in *Shank3* KO mice compared with WT mice (Figures 5G,H, WT total spine density: 12.46 ± 1.29/10 μm, *n* = 18 dendrites from three mice, KO total spine density: 7.64 ± 0.53/10 μm, *n* = 20 dendrites from three mice, two-tailed unpaired separate variance estimation *t*-test, *p* = 0.002). Among the different spine types, the density of mushroom, stubby, and filopodia spines was reduced, but not that of the long thin spines (Figures 5G,I and Supplementary Figures S6D–F, mushroom spine density, WT: 1.53 ± 0.20/10 μm, *n* = 17 dendrites from three mice, KO: 0.45 ± 0.06/10 μm, *n* = 20 dendrites from three mice, Mann–Whitney *U*-test, *p* < 0.0001; stubby spine density, WT: 1.81 ± 0.24/10 μm, *n* = 18 dendrites from three mice, KO: 3.91 ± 0.27/10 μm, *n* = 20 dendrites from three mice, *p* < 0.0001; long thin spine density, WT: 1.96 ± 0.57/10 μm, *n* = 18 dendrites from three mice, KO: 1.25 ± 0.18/10 μm, *n* = 20 dendrites from three mice, Mann–Whitney *U*-test, *p* = 0.86; filopodia spine density, WT: 6.91 ± 1.24/10 μm, *n* = 18 dendrites from three mice, KO: 2.03 ± 0.40/10 μm, *n* = 20 dendrites from three mice, Mann–Whitney *U*-test, *p* = 0.001). In addition, we also analyzed the basal dendrites from pyramidal cells of cortical layers V–VI, which have a large cell body and long dendritic processes. Interestingly, we found that these neurons of *Shank3* KO mice showed more complicated dendrites than those of WT mice (Supplementary Figures S5A–C, WT: *n* = 11 neurons from three mice; KO: *n* = 14 neurons from three mice, Friedman’s *M* test, *p* < 0.0001). Increased intersections were found from 100 to 120 μm in *Shank3* KO mice compared with WT mice (Sholl radius in 100 μm, WT: 11.36 ± 1.94, KO: 17.86 ± 2.05, *p* = 0.03; Sholl radius in 110 μm, WT: 8.91 ± 1.48 KO: 14 ± 1.83, *p* < 0.05; Sholl radius in 120 μm, WT: 6 ± 1.18, KO: 10.71 ± 1.48, *p* = 0.03). However, there were no changes in dendritic length or volume between *Shank3* KO and WT mice (Supplementary Figures S5D,E, dendritic length, WT: 3001 ± 308.3 μm, *n* = 11 neurons from three mice, KO: 3081 ± 246.9 μm, *n* = 14 neurons from three mice, *p* = 0.84; dendritic volume, WT: 2826 ± 592.7 μm^3^, *n* = 11 neurons from three mice, KO: 5109 ± 1138 μm^3^, *n* = 14 neurons from three mice, *p* = 0.27).

**FIGURE 6:**
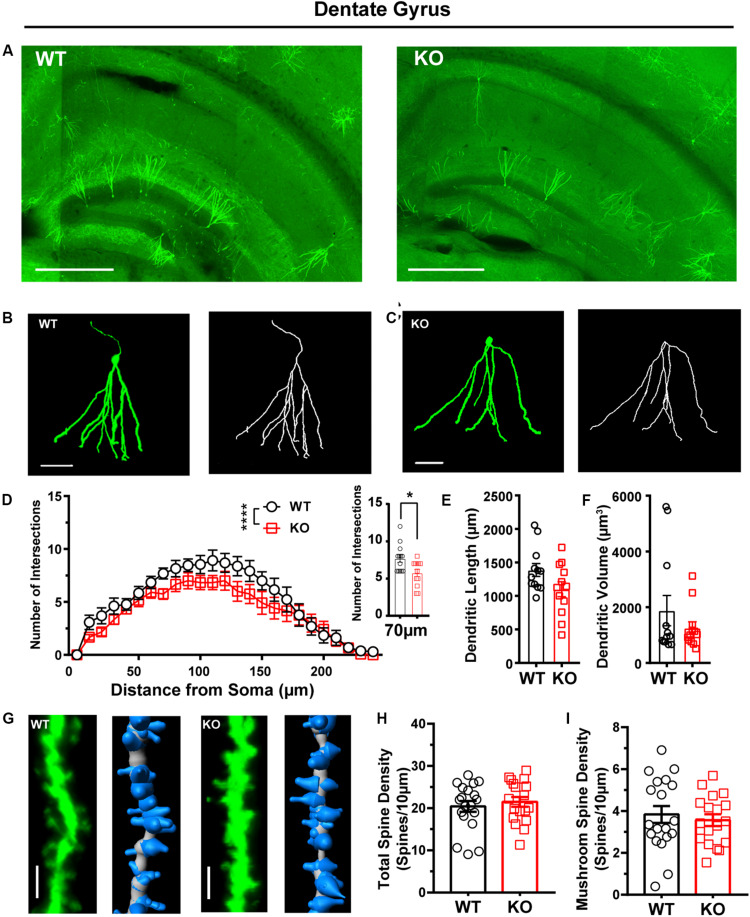
Dendritic complexity was reduced, but dendritic spine density was relatively normal in the dentate granular neurons from the hippocampus of *Shank3* KO mice. **(A)** Representative images showing the distribution of labeled neurons in the dentate gyrus (DG) of WT and KO mice. Scale bar: 500 μm. Representative projection neurons and reconstructed images in the DG of WT **(B)** and KO mice **(C)**. Scale bar: 50 μm. **(D)** Sholl analysis showed that the dendritic complexity of DGs in KO mice was similar to that in WT mice, and specific differences existed between WT and KO mice in Sholl radius (WT: *n* = 13 neurons from three mice, KO: *n* = 12 neurons from three mice, Friedman’s *M* test, χ^2^ = 24.35, *df* = 1, *p* < 0.0001; Sholl radius in 70 μm, WT: 7.69 ± 0.51, KO: 5.75 ± 0.49, two-tailed unpaired *t*-test, *t* = 2.72, *df* = 23, *p* = 0.01). **(E)** The dendritic lengths of dentate granular neurons were similar between KO mice and WT mice (WT: 1387 ± 96.76 μm, *n* = 12 neurons from three mice; KO: 1101 ± 112.4 μm, *n* = 12 neurons from three mice; two-tailed unpaired *t*-test, *t* = 1.94, *df* = 22, *p* = 0.07). **(F)** The dendritic volume of dentate granular neurons was similar between KO mice and WT mice (WT: 1879 ± 541.2 μm^3^, *n* = 12 neurons from three mice; KO: 1253 ± 224.3 μm^3^, *n* = 12 neurons from three mice; Mann–Whitney *U*-test, *Z* = 0.000, *p* = 1.00). **(G)** Representative spine and reconstructed images in the hippocampus of WT (left) and KO mice (right). Scale bar: 2 μm. **(H)** The total spine density was similar between *Shank3* KO mice and WT mice (WT: 20.40 ± 1.29/10 μm, *n* = 19 dendrites from three mice; KO: 21.49 ± 1.17/10 μm, *n* = 18 dendrites from three mice; two-tailed unpaired *t*-test, *t* = -0.62, *df* = 35, *p* = 0.54). **(I)** The mushroom spine density was similar between KO mice and WT mice (WT: 3.8 ± 0.40/10 μm, *n* = 19 dendrites from three mice; KO: 3.56 ± 0.28/10 μm, *n* = 18 dendrites from three mice; two-tailed unpaired *t*-test, *t* = 0.55, *df* = 35, *p* = 0.59). Data are presented as the mean ± the SEM. *p < 0.05, ****p < 0.0001. WT, wild-type mice; KO, *Shank3* KO mice.

In addition to the striatum and cortex, *Shank3* is also expressed in the hippocampus (Peça et al., 2011). Previous work did not find memory-related behavior or neuronal function deficits of the hippocampus in *Shank3* KO mice. Therefore, we investigated the cell structure characteristics of dentate granule cells in WT and *Shank3* KO mice as a negative control (Figures 6A–C). We found that dendritic complexity of *Shank3* KO mice was reduced compared with that of WT mice (Figure 6D, WT: *n* = 13 neurons from three mice, KO: *n* = 12 neurons from three mice, Friedman’s *M* test, *p* < 0.0001). A reduced intersection was found at 70 μm in *Shank3* KO mice compared with WT mice (Sholl radius at 70 μm, WT: 7.69 ± 0.51, KO: 5.75 ± 0.49, *p* = 0.01). There was no difference in dendritic length or volume between *Shank3* KO mice and WT mice (Figures 6E,F, dendritic length, WT: 1387 ± 96.76 μm, *n* = 12 neurons from three mice, KO: 1101 ± 112.4 μm, *n* = 12 neurons from three mice, *p* = 0.07; dendritic volume, WT: 1879 ± 541.2 μm^3^, *n* = 12 neurons from three mice, KO: 1253 ± 224.3 μm^3^, *n* = 12 neurons from three mice, Mann–Whitney *U*-test, *p* = 1.00). The total spine density was similar between *Shank3* KO mice and WT mice (Figures 6G,H, total spine density, WT: 20.40 ± 1.29/10 μm, *n* = 19 dendrites from three mice, KO: 21.49 ± 1.17/10 μm, *n* = 18 dendrites from three mice, *p* = 0.54). Among the different spine types, we found a reduction only in long thin spine density (Figures 6G,I and Supplementary Figures S6G–I, mushroom spine density, WT: 3.8 ± 0.40/10 μm, *n* = 19 dendrites from three mice, KO: 3.56 ± 0.28/10 μm, *n* = 18 dendrites from three mice, *p* = 0.59; stubby spine density, WT: 2.46 ± 0.46/10 μm, *n* = 19 dendrites from three mice; KO: 2.81 ± 0.34/10 μm, *n* = 18 dendrites from three mice, two-tailed unpaired separate variance estimation *t*-test, *p* = 0.55; long thin spine density, WT: 1.20 ± 0.24/10 μm, *n* = 19 dendrites from three mice, KO: 0.50 ± 0.16/10 μm, *n* = 18 dendrites from three mice, Mann–Whitney *U*-test, *p* = 0.01; filopodia spine density, WT: 12.91 ± 0.82/10 μm, *n* = 19 dendrites from three mice, KO: 14.62 ± 1.21/10 μm, *n* = 18 dendrites from three mice, *p* = 0.25). In addition to dentate granular cells, we also compared the dendritic processes of pyramidal neurons in the CA1 of the hippocampus between *Shank3* KO mice and WT mice and did not find significant differences, including the Sholl intersections, dendritic length, and volume (Supplementary Figures S5F–J, Sholl intersections WT: *n* = 11 neurons from three mice; KO: *n* = 7 neurons from three mice, Friedman’s *M* test, *p* = 0.08; dendritic length, WT: 5793 ± 1313 μm, *n* = 13 neurons from three mice, KO: 5757 ± 1775 μm, *n* = 7 neurons from three mice, *p* = 0.61; dendritic volume, WT: 2836 ± 226.5 μm^3^, *n* = 13 neurons from three mice, KO: 3660 ± 418.5 μm^3^, *n* = 7 neurons from three mice, *p* = 0.07). In addition to the morphological changes of EGFPf-labeled neurons in *Shank3* KO mice, we found that the number of EGFPf-labeled neurons was different between *Shank3* KO:CaMKIIα-Cre mice and their littermate controls. We counted the EGFP-labeled neurons in the striatum and cortex in those mice. The number of EGFPf-labeled neurons in the striatum was greater in WT mice than in KO mice (Supplementary Figure S7A, WT: 56.5 ± 3.84, *n* = 12 slice dendrites from three mice, KO: 34.17 ± 2.01, *n* = 12 slices from three mice, *p* < 0.0001). The number of EGFPf-labeled neurons in the sensory and motor cortex was higher in WT mice than in KO mice (Supplementary Figure S7B, WT: 40.67 ± 2.21, *n* = 12 slice dendrites from three mice, KO: 25.92 ± 0.85, *n* = 12 slices from three mice, *p* < 0.0001).

Overall, we confirmed that the FLEX-EGFPf switch system can successfully label single neurons in Cre transgenic mice, and this method could also be used to visualize the morphological traits of single neurons in autistic mouse models.

## Discussion

In this study, we established a FLEX-EGFPf switch system to sparsely label the specific types of neurons throughout the whole brain of Cre transgenic mice using systematic AAV injection. We verified the labeling effect and the specificity of this strategy both *in vitro* and *in vivo*. Furthermore, we applied this method to investigate the dendritic process complexity and dendritic spine density of projection neurons in various brain areas in an autistic mouse model, *Shank3* KO mice. The results suggested that the projection neurons in different brain regions of *Shank3* KO mice showed diverse neuronal morphological deficits in the striatum, cortex, and hippocampus. Our method provided a new straightforward approach to screen the morphological changes of specific types of neurons throughout the whole brain. It can be used not only for ASD mouse models but also for other psychiatric disorder and neurological disease animal models.

There are several strategies for sparse labeling of specific types of neurons in the brain. Filling dye into a certain type of neuron-expressed fluorescent protein is an easy way to implement this requirement (Ren et al., 2017). This method can be used to visualize neuronal morphology after electrophysiological recording. This method can also be applied *in vivo* combined with a two-photon imaging system. However, the efficiency of this method is relatively low, and it cannot label the specific types of neurons throughout the whole brain. Recently, more genetic methods have been used to label cell-type-specific neurons in the brain, such as mosaicism with a repeat frameshift strategy (Lu and Yang, 2017) and stochastic gene activation with a regulated sparseness strategy (Ibrahim et al., 2018). Though it is powerful to use those methods for labeling neurons in mouse brains, the generation of new mouse lines is time-consuming. There are also dual-virus systems to accomplish cell-type-specific labeling (Zhu et al., 2014; Lin et al., 2018). Compared with those methods, our current strategy is simple and straightforward. First, for cell type specificity, we used the Cre-loxP system, which has already been demonstrated to be a mature and straightforward strategy. Our AAV-hSyn-DIO-EGFP-P2A-EGFPf vector could potentially be used for most Cre transgenic mouse lines. This method could also achieve cell-type-specific labeling when combined with a specific promoter-driven Cre virus. This study will streamline experiments for the study of neuronal morphological changes in mouse models of psychiatric disorders and neurological diseases. Second, the labeling of sparseness and stochasticity is simple and controllable through the adjustment of the viral titer in our method. Third, we selected EGFPf as the reporter protein to visualize the neurons to achieve fine process labeling. This fluorescence reporter has been proven to be a good indicator for dendritic spine labeling (Cai et al., 2013; Zhang Q. et al., 2016; Wang et al., 2017; Guo et al., 2019). Fourth, our virus delivery method is uncomplicated and suitable for mice from P0 to adulthood. However, since it takes 3 weeks to obtain good expression of EGFPf in our method, it is hard for the morphological characteristics of the early neurodevelopmental study of ASD models with such long expression times. This is quite important, particularly for neurodevelopmental disorders, such as ASD. To conquer this problem, we will try to use *in utero* viral injection to apply this method in visualizing neurons during the postnatal stages.

Autism spectrum disorder is a neurodevelopmental disease that is characterized by social dysfunction and repetitive behaviors (Willsey and State, 2015). Though the causes of ASD are still unclear, extensive genetic studies have suggested that genetic abnormalities contribute to the etiology of ASD. *Shank3* is an ASD risk gene that has been proven by human genetic studies and animal models (Monteiro and Feng, 2017). SHANK3 is a scaffolding protein that involves a cytoskeleton-associated signaling complex at the postsynaptic density of excitatory synapses in the mammalian brain (Naisbitt et al., 1999; Sheng and Kim, 2000). Several groups generated different *Shank3* KO mouse lines and confirmed that those mouse lines showed social interaction deficits and overgrooming, which are similar to the phenotypes observed in ASD patients. These studies suggested that these mouse lines are promising mouse models for ASD research (Bozdagi et al., 2010; Peça et al., 2011; Wang et al., 2011; Kouser et al., 2013; Lee et al., 2015; Speed et al., 2015; Jaramillo et al., 2016; Mei et al., 2016; Wang et al., 2016; Zhou et al., 2016). Among the *Shank3* KO mouse lines, we chose *Shank3B* KO in our study (Peça et al., 2011). In the initial characterization of the neuronal morphology of MSNs in *Shank3B* KO mice, Peça et al. (2011) found that young (4-week) *Shank3B* KO mice showed an increased dendritic complexity and total dendritic length of MSNs compared with the MSNs in WT mice. Interestingly, we labeled the MSNs in the striatum of *Shank3B* KO:CaMKIIα-Cre mice and found no difference in total dendritic length but decreased dendritic complexity. We speculate that the major reason for these inconsistent results might be the mouse age difference between our study and that of Peça and colleagues. We used older (10-week-old) mice than the younger mice (4-week-old) used in the previous study. A previous clinical study showed that brain size in autistic patients was slightly reduced at birth, dramatically increased within the first year of life, but then plateaued so that the majority of patients were within the normal range by adulthood (Redcay and Courchesne, 2005). The discrepancy in the total dendritic length and dendritic complexity between the previous study and our results might capture the different stages of the MSN dendritic changes in *Shank3B* KO mice. For dendritic spine density, both Peça’s results and our results showed a significant reduction in *Shank3B* KO compared with WT animals. Additionally, reports from other laboratories (Bozdagi et al., 2010; Wang et al., 2011; Kouser et al., 2013) showed a similar reduction of dendritic spines in other *Shank3* KO mice. These results suggested that dendritic spine changes might be a key morphological deficit in *Shank3* KO mice. In addition, we also analyzed the dendritic processes and spines of pyramidal neurons in the cortex in *Shank3B* KO:CaMKIIα-Cre mice. The results showed a significant reduction in both dendritic complexity and spine density of layer II–III neurons in *Shank3B* KO mice compared with littermate controls. Interestingly, we found that *Shank3B* KO mice showed even more complicated dendrites in layer V–VI pyramidal neurons than WT mice. This result indicated that *Shank3B* insufficiency caused heterogeneous changes in the cortex. Further study is needed to determine the consequences of changes in animal behavior caused by these heterogeneous changes. In addition, we found that the number of EGFPf-labeled neurons was less in *Shank3* KO:CaMKIIα-Cre mice than in WT:CaMKIIα-Cre mice. One of the possible reasons is the reduction of projection neurons in *Shank3* KO mice compare with those in WT animals. And also, SHANK3 and CaMKIIα are two extremely abundant proteins in the postsynaptic densities of excitatory synapses. A recent study showed that SHANK3 interacts with CaMKIIα through its N-terminal domain. The lack of SHANK3 might cause changes in the function and distribution of CaMKIIα (Perfitt et al., 2020). We speculate that the fewer number of EGFPf-labeled neurons might be caused by these two reasons.

## Conclusion

In summary, we developed a simple and straightforward strategy to perform sparse cell-type-specific neuronal labeling throughout the whole brain. Moreover, we used this strategy to analyze the dendritic complexity and spine density of the projection neurons in the striatum, cortex, and hippocampus in *Shank3* KO mice. We found heterogeneous morphological deficits of single neurons in this ASD mouse model. Our method has many potential implications for future understanding of the cell-type-specific changes that occur in not only ASD but also other psychiatric disorders and neurological diseases.

## Data Availability Statement

The datasets generated during the current study are available from the corresponding author on reasonable request.

## Ethics Statement

The animal study was reviewed and approved by the Institutional Animal Care and Use Committee of the Fourth Military Medical University.

## Author Contributions

QC and WW conceived the project, designed the experiments, and prepared the manuscript based on the draft by DC. DC and KR performed the experiments and analyzed the data. HL analyzed the data. HMa performed statistical analyses. KR, ZL, HMo, SX, and YS revised it critically for important intellectual content. All authors approved the final version of the manuscript submitted for publication, all persons designated authors qualify for authorship, and all those who qualify for authorship are listed.

## Conflict of Interest

The authors declare that the research was conducted in the absence of any commercial or financial relationships that could be construed as a potential conflict of interest.
