# Supplementary material for: A Whole-Brain Cell-Type-Specific Sparse Neuron Labeling Method and Its Application in a Shank3 Autistic Mouse Model
Source: Front Cell Neurosci. 2020 Jun 5;14:145. doi: 10.3389/fncel.2020.00145 (PMC7291601; doi:10.3389/fncel.2020.00145)
Supplement: Supplementary file 1 [file Table_1.docx]

Supplementary Material

# Supplementary Figures and Tables

## Supplementary Figures

#
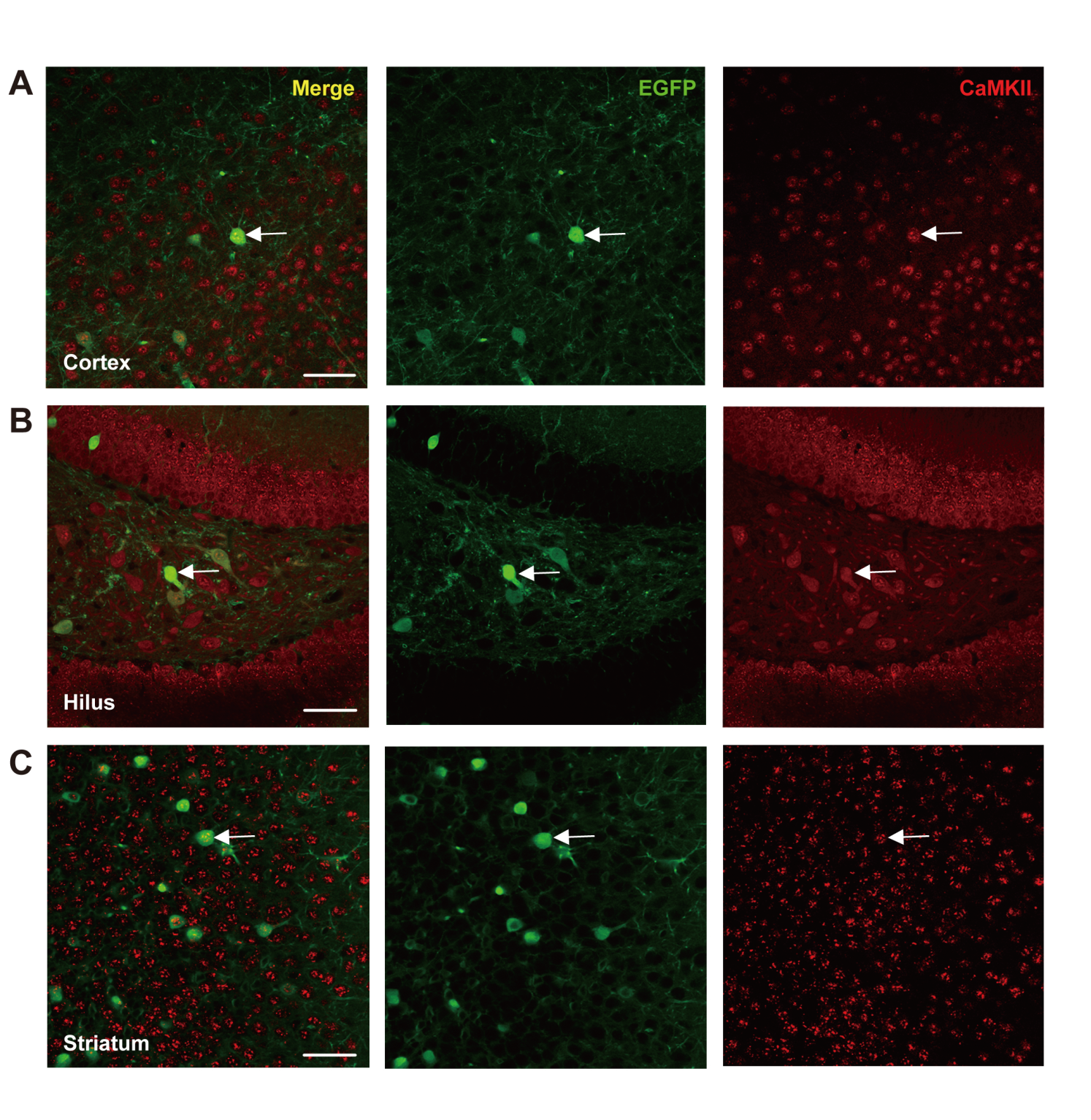


# Supplementary Figure 1. The specificity of Cre-dependent expression of EGFP neuron in the CaMKIIα-Cre transgenic mice. Double immunostaining for the EGFP positive neurons (Green) and CaMKIIα (red) in cortex (A), Hilus in dentate gyrus (B) and striatum (C). The arrow indicates EGFP and CaMKII colocalized at the same neuron. Scale bar: 50 μm.

#
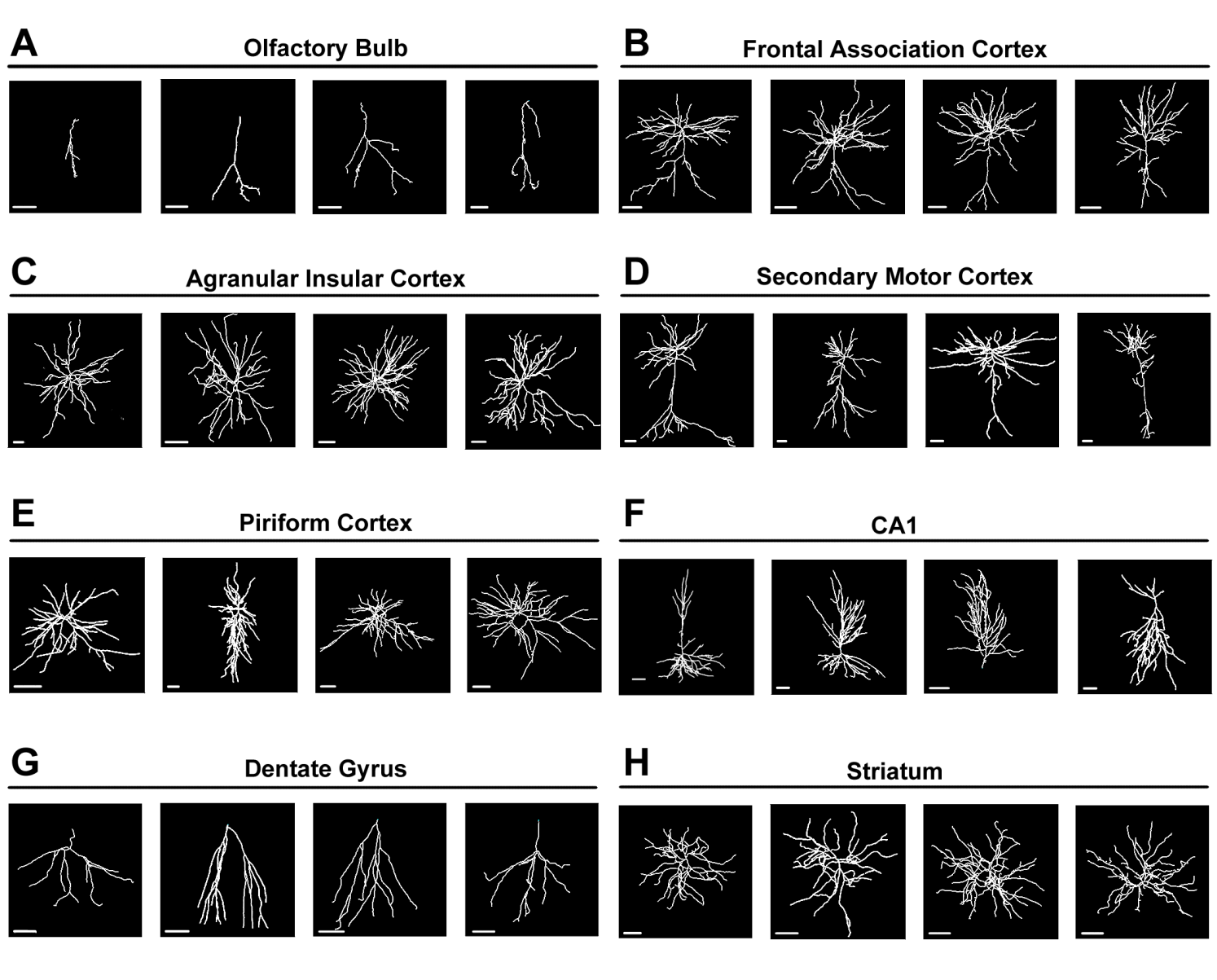


# Supplementary Figure 2. The reconstructed images of single neurons in different brain areas in CaMKIIα-Cre transgenic mice. (Neurons used for the Sholl analysis in Figure 3) Scale bar: 50 μm.

#
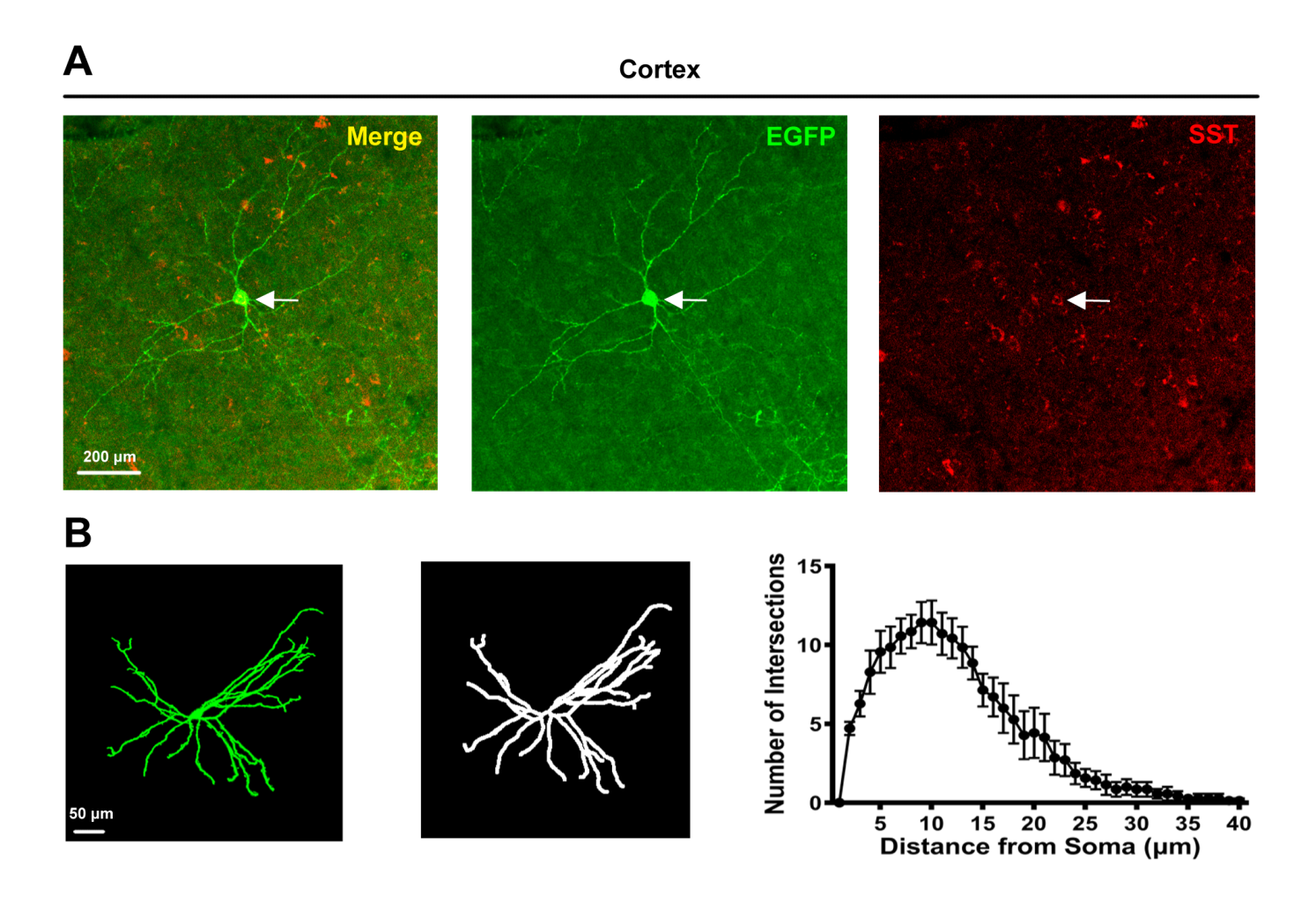


# Supplementary Figure 3. Somatostatin (SST) interneuron can be sparsely labeled in SST-Cre transgenetic mice by AAV -hSyn-DIO-P2A-EGFPf. (A) The specificity of Cre-dependent expression of EGFP neuron in the SST-Cre transgenic mice. Double immunostaining of EGFP (Green) and somatostatin (Red) for the EGFP positive neurons in cortex. The arrows indicate that EGFP and SST co-localized at the same neuron. (B) Left: a representative image of EGFP labeled neuron and its reconstructed image; Right: the Sholl analysis results of EGFP-labeled neurons of cortex in SST-Cre transgenic mice (*n* = 5 neurons from 3 mice).

#
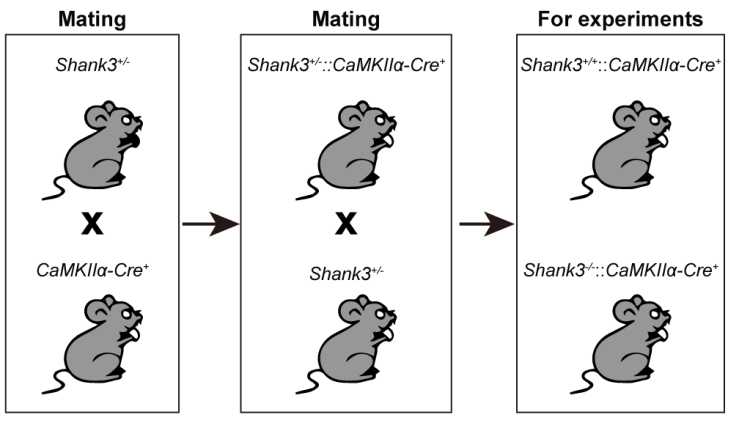


# Supplementary Figure 4. Breeding strategy used in this study.

#
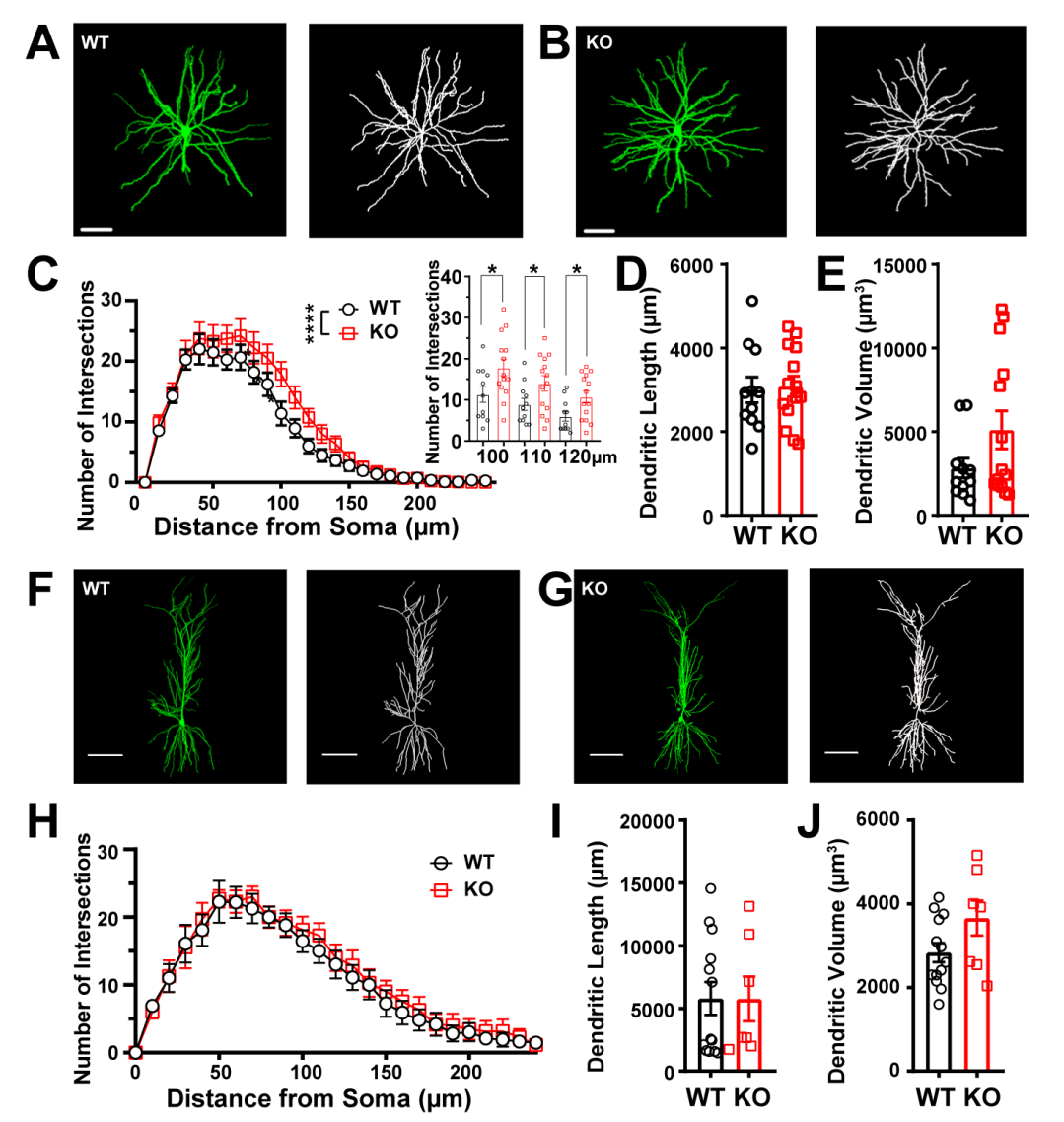


**Supplementary Figure 5. The dendritic complexity and spine density changes of neurons in layer V-VI cortex and hippocampus CA1 of *Shank3* KO mouse.** **(A-B)** Representative projection neurons and reconstruction images in the cortical layer V-VI of WT **(A)** and *Shank3* KO mice **(B)**. Scale bar: 50 μm. **(C)** The Sholl analysis showed that the dendritic complexity of pyramidal neurons in *Shank3* KO mice was reduced compared to that of WT mice (WT: *n* = 11 neurons from 3 mice; KO: *n* = 14 neurons from 3 mice. Friedman's M test , χ^2^ = 18.31, df = 1, *p* <0.0001) and inset showed that specific differences exist between WT and KO mice in Sholl radius (the Sholl radius in 100 μm, WT: 11.36 ± 1.94, KO: 17.86 ± 2.05, Two-tailed unpaired *t*-test, t = 2.25, df = 23,*p* = 0.03; the Sholl radius in 110 μm, WT: 8.91 ± 1.48 KO: 14 ± 1.83, Two-tailed unpaired *t*-test, t = 2.08, df = 23, *p* < 0.05 ; the Sholl radius in 120 μm, WT: 6 ± 1.18, KO: 10.71 ± 1.48, Two-tailed unpaired *t*-test, t = 2.40, df = 23, *p* = 0.03). **(D)** The dendritic length of pyramidal neurons were similar between KO mice and WT mice (WT: 3001 ± 308.3 μm, *n* = 11 neurons from 3 mice; KO: 3081± 246.9 μm, *n* = 14 neurons from 3 mice. Two-tailed unpaired *t*-test, t = -0.21, df = 23, *p* = 0.84). **(E)** The dendritic volume of pyramidal neurons were similar between *Shank3* KO mice and WT mice (WT: 2826 ± 592.7 μm^3^, *n* = 11 neurons from 3 mice; KO: 5109 ± 1138 μm^3^, *n* = 14 neurons from 3 mice. Mann-Whitney U test, Z = -1.10, *p* = 0.27). **(F-G)** Representative projection neurons and reconstruction images in the CA1 of WT **(F)** and KO mice **(G)**. Scale bar: 100 μm. **(H)** The Sholl analysis showed that the dendritic complexity of CA1 in *Shank3* KO mice were similar to WT mice (WT: *n* = 11 neurons from 3 mice; KO: *n* = 7 neurons from 3 mice. Friedman's M test, χ^2^ = 3.14, df =1, *p* = 0.08). **(I)** The dendritic length of labeled neurons were similar between *Shank3* KO mice and WT mice (WT: 5793 ±1313 μm, n = 13 neurons from 3 mice; KO: 5757 ±1775 μm, *n* = 7 neurons from 3 mice. Mann-Whitney U test, Z = -0.52, *p* = 0.61). **(J)** The dendritic volume of projection neurons was similar between KO mice and WT mice (WT: 2836 ± 226.5 μm^3^, n = 13 neurons from 3 mice; KO: 3660 ±418.5 μm^3^, *n* = 7 neurons from 3 mice. Two-tailed unpaired *t*-test, t = -1.90, df = 18, *p* = 0.07). Data are presented as the mean ± s.e.m. **p* < 0.05, *****p* < 0.0001. WT: wild type mice, KO: *Shank3* KO mice.


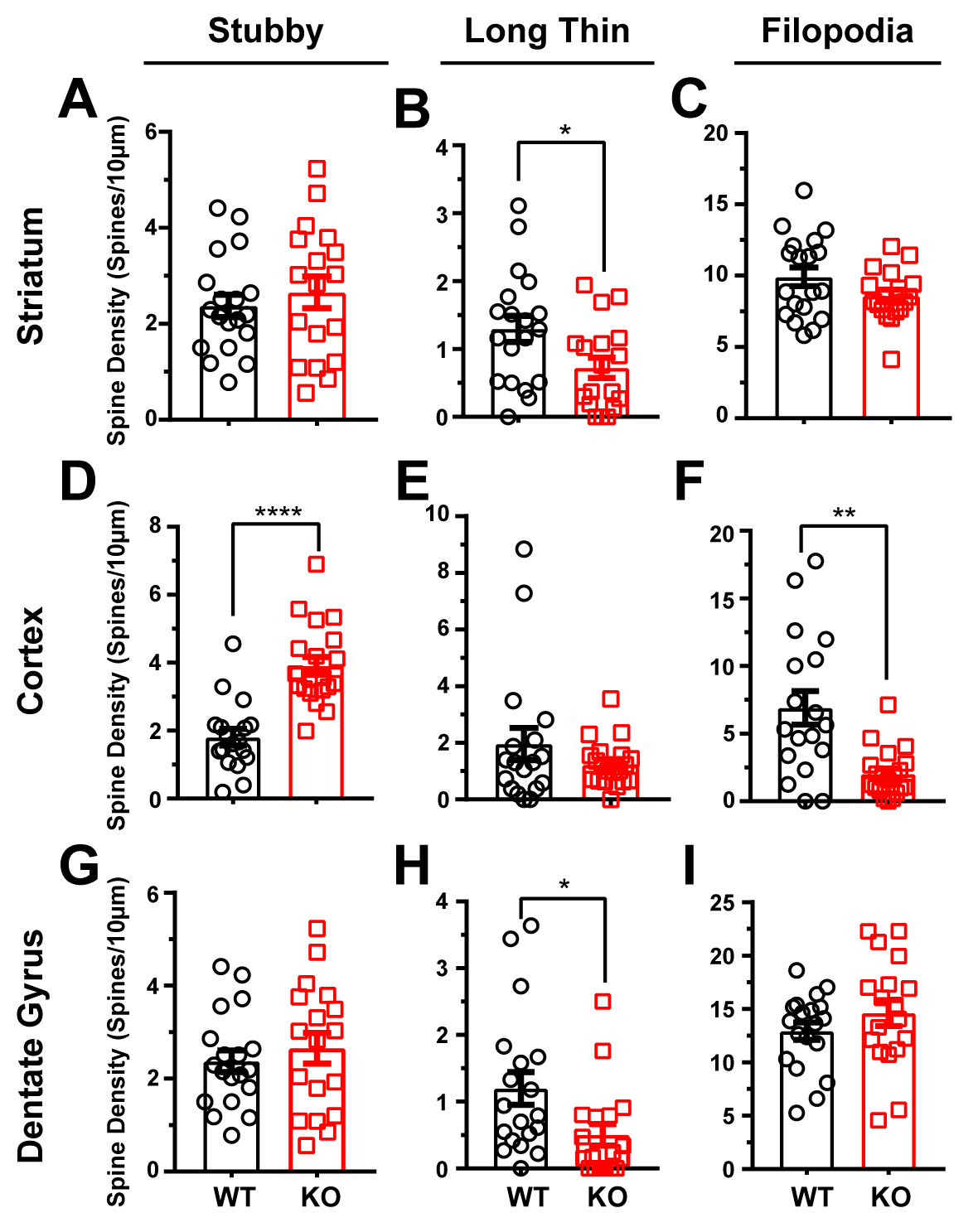


**Supplementary Figure 6. Comparison of dendritic spine density in different brain regions in WT mice and *Shank3* KO mice. (A-C)** Statistical results of different spines in the striatum of WT and Shank3 KO mice, (**A**) The stubby spine density similar between *Shank3* KO mice and WT mice (WT: 2.37 ± 0.23/10 μm, *n* = 19 dendrites from 3 mice; KO: 2.65 ± 0.33/10 μm, *n* = 18 dendrites from 3 mice. Two-tailed unpaired *t*-test, t = -0.70, df = 35, *p* = 0.49). **(B)** The density of long thin reduced in *Shank3* KO mice compared with WT mice (WT: 1.30 ± 0.19/10 μm, *n* = 19 dendrites from 3 mice; KO: 0.72 ± 0.15/10 μm, *n* = 18 dendrites from 3 mice. Two-tailed unpaired *t*-test, t = 2.33, df = 35, *p* = 0.03). **(C)** The filopodia spine density similar between *Shank3* KO mice and WT mice. (WT: 9.91 ± 0.66/10 μm, *n* = 19 dendrites from 3 mice; KO: 8.59 ± 0.43/10 μm, n = 18 dendrites from 3 mice. Two-tailed unpaired separate variance estimation *t*-test,t = 1.68, df = 30.65 , *p* = 0.10). **(D-F)** Statistical results of different spines in the cortex of WT and *Shank3* KO mice. **(D)** The density of stubby increased in *Shank3* KO mice compared with WT mice (WT: 1.81 ± 0.24/10 μm, *n* = 18 dendrites from 3 mice; KO: 3.91 ± 0.27/10 μm, *n* = 20 dendrites from 3 mice. Two-tailed unpaired *t*-test, t = -5.83, df = 36, *p* < 0.0001). **(E)** The long thin spine density similar between *Shank3* KO mice and WT mice (WT: 1.96 ± 0.57/10 μm, n = 18 dendrites from 3 mice, KO: 1.25 ± 0.18/10 μm, *n* = 20 dendrites from 3 mice. Mann-Whitney U test, Z = -0.18, *p* = 0.86). **(F)** The density of filopodia reduced in *Shank3* KO mice compared with WT mice (WT: 6.91 ± 1.24/10 μm, *n* = 18 dendrites from 3 mice; KO: 2.03 ± 0.40/10 μm, *n* = 20 dendrites from 3 mice. Mann-Whitney U test, Z = -3.25, *p* = 0.001). **(G-I)** Statistical results of different spines in the CA1 (field CA1 of hippocampus) of WT and *Shank3* KO mice. (**G**) The stubby spine density similar between *Shank3* KO mice and WT mice (WT: 2.46 ± 0.46/10 μm, *n* = 19 dendrites from 3 mice; KO: 2.81 ± 0.34/10 μm, *n* = 18 dendrites from 3 mice. Two-tailed unpaired separate variance estimation *t*-test, t = -0.61, df = 32.38, *p* = 0.55). **(H)** The density of long thin reduced in *Shank3* KO mice compared with WT mice. (WT: 1.20 ± 0.24/10 μm, *n* = 19 dendrites from 3 mice; KO: 0.50 ± 0.16/10 μm, *n* = 18 dendrites from 3 mice. Mann-Whitney U test, Z = -2.59, *p* = 0.01). **(I)** The filopodia spine density similar between *Shank3* KO mice and WT mice (WT: 12.91 ± 0.82/10 μm, *n* = 19 dendrites from 3 mice; KO: 14.62 ± 1.21/10 μm, *n* = 18 dendrites from 3 mice. Two-tailed unpaired *t*-test, t = -1.18, df = 35, *p* = 0.25); Data are presented as the mean ± s.e.m. **p* < 0.05, ***p* < 0.01, *****p* < 0.0001.WT: wild type mice, KO: *Shank3* KO mice.


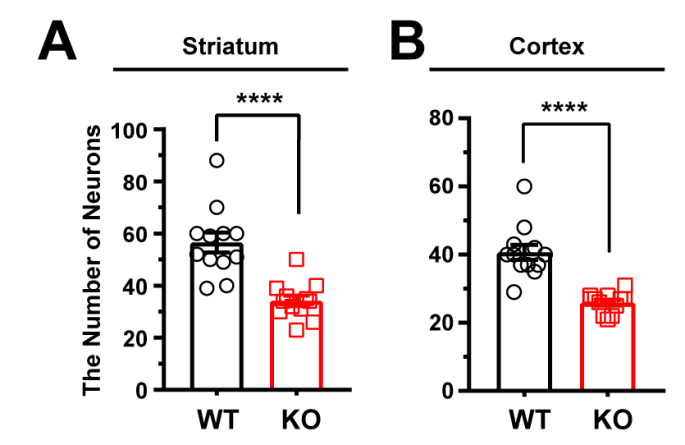


**Supplementary Figure 7. The number of labeled neurons in different regions of WT and *Shank3* KO mice. (A)** The number of EGFP-labeled neurons in the striatum was more in WT mice than in *Shank3* KO mice (WT: 56.5 ± 3.84, n = 12 slices from 3 mice; KO:34.17 ± 2.01, *n* = 12 slices from 3 mice. Two-tailed unpaired *t*-test, t = 5.15, df = 22, *p* <0.0001). **(B)** The number of EGFP-labeled neurons in the sensory and motor cortex was more in WT mice than in *Shank3* KO mice (WT: 40.67 ± 2.21, *n* = 12 slices from 3 mice; KO: 25.92 ± 0.85, *n* = 12 slices from 3 mice. Two-tailed unpaired *t*-test, t = 6.23, df = 22, *p* < 0.0001). Data are presented as the mean ± s.e.m. *****p* < 0.0001.WT: wild type mice, KO: *Shank3* KO mice.

## 2. Supplementary Tables

**Supplementary Table 1. The antibody information in the figures.**

| **Figure** | **Antibody information** | **Antibody concentration** | **Cat. NO.** | **Company** |
| --- | --- | --- | --- | --- |
| **Fig. 2-6,**  **Suppl Fig. 5** | Rabbit anti-GFP | 1:1000 | A11122 | Invitrogen, USA |
|  | Alexa Fluor 488-conjugated donkey anti-rabbit IgG | 1:1000 | A32790 | Invitrogen, USA |
| **Suppl Fig. 1** | Mouse anti-GFP | 1:200 | ab1218 | abcam, USA |
|  | Alexa Fluor 488-conjugated donkey Mouse IgG | 1:1000 | ab150117 | abcam, USA |
|  | Rabbit anti-CaMKII | 1:200 | ab5683 | abcam, USA |
|  | Alexa Fluor 594-conjugated donkey anti-rabbit IgG | 1:1000 | ab150132 | abcam, USA |
| **Suppl Fig. 3** | Rabbit anti-GFP | 1:1000 | A11122 | Invitrogen, USA |
|  | Alexa Fluor 488-conjugated donkey anti-rabbit IgG | 1:1000 | A32790 | Invitrogen, USA |
|  | Rat anti-Somatostatin | 1:100 | MAB354 | Sigma-Aldrich, USA |
|  | Alex Fluor 594-conjugated donkey anti-rat IgG | 1:1000 | ab150160 | abcam, USA |

**Supplementary Table 2. The particular statistical methods used in the figures.**

| **Experimental design** | **Group** | **Factor** | **Normality Test (Shapiro-Wilk)** | **Homogeneity of variance test (Levene’s test)** | **Hypothesis test** |
| --- | --- | --- | --- | --- | --- |
| Completely random design | 2 groups | 1 | *p* > 0.05, normal distribution | *p*>0.05, meet homogeneity of variance | Two-tailed unpaired *t*-test |
|  |  |  |  | *p*<0.05, not meet homogeneity of variance | Two-tailed unpaired separate variance estimation t-test |
|  |  |  | *p* < 0.05, not normal distribution | meet or not meet homogeneity of variance | Mann-Whitney U test |
| Repeated measurement design | ≥2 groups | 2 | *p* < 0.05, not normal distribution | *p*<0.05, not meet homogeneity of variance | Friedman's M test and Student-Newman-Keuls test |

**Supplementary Table 3. The detail of statistical results in the figures.**

| **Data** | **Response variable** | **Groups** | **n define as** | **Normality Test (Shapiro-Wilk)** | **Homogeneity of variance test (Levene’s test)** | **Hypothesis test** | **Test value** | **P value** |
| --- | --- | --- | --- | --- | --- | --- | --- | --- |
|  |  |  |  |  |  |  |  |  |
| **Figure 4D** | No.of intersection | WT=44 | neuron | Not all subgroups meets p＞0.05 | Not all subgroups meets p＞0.05 | Friedman's M test | Χ^2^=7.750 df=1 | *p*=0.005 |
|  |  | KO=37 |  |  |  |  |  |  |
| **Figure 4E** | Dendritic length | WT=40 | neuron | W=0.960 df=40 p=0.164 | F=7.102 df1=1 df2=74 p=0.009 | Two-tailed unpaired separate variance estimation t-test | t=1.620 df=69.839 | *p*=0.110 |
|  |  | KO=36 |  | W=0.978 df=36 *p=*0.678 |  |  |  |  |
| **Figure 4F** | Dendritic volume | WT=40 | neuron | W=0.808 df=40 *p<*0.0001 | F=2.875 df1=1 df2=75 *p=*0.094 | Mann-Whitney U test | Z=-0.907 | *p*=0.364 |
|  |  | KO=37 |  | W=0.646 df=37 *p<*0.0001 |  |  |  |  |
| **Figure 4H** | Total spine density | WT=19 | dendrite | W=0.966 df=19 *p=*0.686 | F=0.330 df1=1 df2=35 *p=*0.569 | Two-tailed unpaired t-test | t=2.437 df=35 | *p*=0.020 |
|  |  | KO=18 |  | W=0.973 df=18 *p=*0.849 |  |  |  |  |
| **Figure 4I** | mushroom density | WT=19 | dendrite | W=0.964 df=19 *p=*0.662 | F=0.082 df1=1 df2=35 *p=*0.776 | Two-tailed unpaired t-test | t=2.468 df=35 | *p*=0.019 |
|  |  | KO=18 |  | W=0.977 df=18 *p=*0.918 |  |  |  |  |
| **Figure 5D** | No.of intersection | WT=17 | neuron | Not all subgroups meets *p>*0.05 | Not all subgroups meets *p>*0.05 | Friedman's M test | Χ^2^=12.634 df=1 | *p*<0.0001 |
|  |  | KO=17 |  |  |  |  |  |  |
| **Figure 5E** | Dendritic length | WT=16 | neuron | W=0.984 df=16 *p=*0.988 | F=7.291 df1=1 df2=32 *p=*0.011 | Two-tailed unpaired separate variance estimation t-test | t=1.131 df=22.158 | *p*=0.270 |
|  |  | KO=18 |  | W=0.988 df=18 *p=*0.995 |  |  |  |  |
| **Figure 5F** | Dendritic volume | WT=16 | neuron | W=0.960 df=16 *p=*0.656 | F=0.738 df1=1 df2=32 *p=*0.397 | Mann-Whitney U test | Z=-1.277 | *p*=0.202 |
|  |  | KO=18 |  | W=0.766 df=18 *p=*0.001 |  |  |  |  |
| **Figure 5H** | Total spine density | WT=18 | dendrite | W=0.930 df=18 *p=*0.192 | F=10.343 df1=1 df2=36 *p=*0.003 | Two-tailed unpaired separate variance estimation t-test | t=3.458 df=22.707 | *p*=0.002 |
|  |  | KO=20 |  | W=0.909 df=20 *p=*0.061 |  |  |  |  |
| **Figure 5I** | mushroom density | WT=17 | dendrite | W=0.921 df=17 *p=*0.153 | F=33.573 df1=1 df2=35 *p<*0.0001 | Mann-Whitney U test | Z=-4.085 | *p*<0.0001 |
|  |  | KO=20 |  | W=0.882 df=20 *p=*0.019 |  |  |  |  |
| **Figure 6D** | No.of intersection | WT=13 | neuron | Not all subgroups meets *p>*0.05 | Not all subgroups meets *p>*0.05 | Friedman's M test | Χ^2^=24.351 df=1 | *p*<0.0001 |
|  |  | KO=12 |  |  |  |  |  |  |
| **Figure 6E** | Dendritic length | WT=12 | neuron | W=0.882 df=12 *p=*0.092 | F=0.405 df1=1 df2=22 *p=*0.531 | Two-tailed unpaired t-test | t=1.935 df=22 | *p*=0.066 |
|  |  | KO=12 |  | W=0.981 df=12 *p=*0.987 |  |  |  |  |
| **Figure 6F** | Volume | WT=12 | neuron | W=0.664 df=12 *p<*0.0001 | F=7.808 df1=1 df2=22 *p=*0.011 | Mann-Whitney U test | Z=0.000 | *p*=1.000 |
|  |  | KO=12 |  | W=0.765 df=12 *p=*0.004 |  |  |  |  |
| **Figure 6H** | Total spine density | WT=19 | dendrite | W=0.902 df=19 *p=*0.052 | F=0.019 df1=1 df2=35 *p=*0.891 | Two-tailed unpaired t-test | t=-0.622 df=35 | *p*=0.538 |
|  |  | KO=18 |  | W=0.960 df=18 *p=*0.611 |  |  |  |  |
| **Figure 6I** | mushroom density | WT=19 | dendrite | W=0.955 df=19 *p=*0.479 | F=3.772 df1=1 df2=35 *p=*0.060 | Two-tailed unpaired t-test | t=0.551 df=35 | *p*=0.585 |
|  |  | KO=18 |  | W=0.973 df=18 *p=*0.859 |  |  |  |  |
| **Suppl Fig. 5C** | No.of intersection | WT=11 | neuron | Not all subgroups meets *p>*0.05 | Not all subgroups meets *p>*0.05 | Friedman's M test | Χ^2^=18.305 df=1 | *p*<0.0001 |
|  |  | KO=14 |  |  |  |  |  |  |
| **Suppl Fig. 5D** | Dendritic length | WT=11 | neuron | W=0.929 df=11 *p=*0.405 | F=0.005 df1=1 df2=23 *p=*0.946 | Two-tailed unpaired t-test | t=-0.205 df=23 | *p*=0.839 |
|  |  | KO=14 |  | W=0.948 df=14 *p=*0.526 |  |  |  |  |
| **Suppl Fig. 5E** | Volume | WT=11 | neuron | W=0.783 df=11 *p=*0.006 | F=12.542 df1=1 df2=23 *p=*0.002 | Mann-Whitney U test | Z=-1.095 | *p*=0.274 |
|  |  | KO=14 |  | W=0.800 df=14 *p=*0.005 |  |  |  |  |
| **Suppl Fig. 5G** | No.of intersection | WT=13 | neuron | Not all subgroups meets *p>*0.05 | Not all subgroups meets *p>*0.05 | Friedman's M test | Χ^2^=3.143 df=1 | *p*=0.076 |
|  |  | KO=7 |  |  |  |  |  |  |
| **Suppl Fig. 5H** | Dendritic length | WT=13 | neuron | W=0.834 df=13 *p=*0.018 | F=0.054 df1=1 df2=18 *p=*0.818 | Mann-Whitney U test | Z=-0.515 | *p*=0.606 |
|  |  | KO=7 |  | W=0.824 df=7 *p=*0.071 |  |  |  |  |
| **Suppl Fig. 5I** | Volume | WT=13 | neuron | W=0.942 df=13 *p=*0.482 | F=1.589 df1=1 df2=18 *p=*0.224 | Two-tailed unpaired t-test | t=-1.904 df=18 | *p*=0.073 |
|  |  | KO=7 |  | W=0.860 df=7 *p=*0.152 |  |  |  |  |
| **Suppl Fig. 6A** | Stubby density from striatum | WT=19 | dendrite | W=0.949 df=19 *p=*0.376 | F=3.559 df1=1 df2=35 *p=*0.068 | Two-tailed unpaired t-test | t=-0.699 df=35 | *p*=0.489 |
|  |  | KO=18 |  | W=0.955 df=18 *p=*0.502 |  |  |  |  |
| **Suppl Fig. 6B** | Long thin density from striatum | WT=19 | dendrite | W=0.957 df=19 *p=*0.513 | F=0.558 df1=1 df2=35 *p=*0.460 | Two-tailed unpaired t-test | t=2.329 df=35 | *p*=0.026 |
|  |  | KO=18 |  | W=0.900 df=18 *p=*0.057 |  |  |  |  |
| **Suppl Fig. 6C** | Filopodia density from striatum | WT=18 | dendrite | W=0.900 df=18 *p=*0.057 | F=8.121 df1=1 df2=35 *p=*0.007 | Two-tailed unpaired separate variance estimation t-test | t=1.680 df=30.651 | *p*=0.103 |
|  |  | KO=18 |  | W=0.956 df=18 *p=*0.529 |  |  |  |  |
| **Suppl Fig. 6D** | Stubby density from cortex | WT=18 | dendrite | W=0.930 df=18 *p=*0.197 | F=0.632 df1=1 df2=36 *p=*0.432 | Two-tailed unpaired t-test | t=-5.829 df=36 | *p*<0.0001 |
|  |  | KO=20 |  | W=0.939 df=20 *p=*0.231 |  |  |  |  |
| **Suppl Fig. 6E** | Long thin density from cortex | WT=18 | dendrite | W=0.728 df=18 *p<*0.0001 | F=6.655 df1=1 df2=36 *p=*0.014 | Mann-Whitney U test | Z=-0.175 | *p*=0.861 |
|  |  | KO=20 |  | W=0.907 df=20 *p=*0.056 |  |  |  |  |
| **Suppl Fig. 6F** | Filopodia density from cortex | WT=18 | dendrite | W=0.938 df=18 *p=*0.273 | F=17.123 df1=1 df2=36 *p<*0.0001 | Mann-Whitney U test | Z=-3.246 | *p*=0.001 |
|  |  | KO=20 |  | W=0.882 df=20 *p=*0.019 |  |  |  |  |
| **Suppl Fig. 6G** | Stubby density from dentate gyrus | WT=19 | dendrite | W=0.906 df=19 *p=*0.062 | F=4.407 df1=1 df2=35 *p=*0.043 | Two-tailed unpaired separate variance estimation t-test | t=-0.612 df=32.381 | *p*=0.545 |
|  |  | KO=18 |  | W=0.941 df=18 *p=*0.297 |  |  |  |  |
| **Suppl Fig. 6H** | Long thin density from dentate gyrus | WT=19 | dendrite | W=0.856 df=19 *p=*0.009 | F=3.094 df1=1 df2=35 *p=*0.087 | Mann-Whitney U test | Z=-2.592 | *p*=0.010 |
|  |  | KO=18 |  | W=0.751 df=18 *p<*0.0001 |  |  |  |  |
| **Suppl Fig. 6I** | Filopodia density from dentate gyrus | WT=19 | dendrite | W=0.946 df=19 *p=*0.334 | F=2.315 df1=1 df2=35 *p*=0.137 | Two-tailed unpaired t-test | t=-1.176 df=35 | *p*=0.248 |
|  |  | KO=18 |  | W=0.957 df=18 *p=*0.550 |  |  |  |  |
| **Suppl Fig. 7A** | Number | WT=12 | slice | W=0.908 df=12 *p=*0.199 | F=2.834 df1=1 df2=22 *p*=0.106 | Two-tailed unpaired t-test | t=5.152 df=22 | *p*<0.0001 |
|  |  | KO=12 |  | W=0.955 df=12 *p*=0.713 |  |  |  |  |
| **Suppl Fig. 7B** | Number | WT=12 | slice | W=0.877 df=12 *p*=0.079 | F=2.748 df1=1 df2=22 *p=*0.112 | Two-tailed unpaired t-test | t=6.231 df=22 | *p*<0.0001 |
|  |  | KO=12 |  | W=0.909 df=12 *p*=0.205 |  |  |  |  |
